# Advancements in incorporating metal ions onto the surface of biomedical titanium and its alloys via micro-arc oxidation: a research review

**DOI:** 10.3389/fchem.2024.1353950

**Published:** 2024-02-22

**Authors:** Xue’e Zhang, Wuchao Zhou, Weihong Xi

**Affiliations:** ^1^ Jiangxi Province Key Laboratory of Oral Biomedicine, School of Stomatology, Jiangxi Medical College, Jiangxi Province Clinical Research Center for Oral Diseases, Nanchang University, Nanchang, China; ^2^ Jiangxi Province Key Laboratory of Oral Biomedicine, The Affiliated Stomatological Hospital, Jiangxi Medical College, Jiangxi Province Clinical Research Center for Oral Diseases, Nanchang University, Nanchang, China

**Keywords:** titanium, micro-arc oxidation, metal ions, performance optimization, biological coating

## Abstract

The incorporation of biologically active metallic elements into nano/micron-scale coatings through micro-arc oxidation (MAO) shows significant potential in enhancing the biological characteristics and functionality of titanium-based materials. By introducing diverse metal ions onto titanium implant surfaces, not only can their antibacterial, anti-inflammatory and corrosion resistance properties be heightened, but it also promotes vascular growth and facilitates the formation of new bone tissue. This review provides a thorough examination of recent advancements in this field, covering the characteristics of commonly used metal ions and their associated preparation parameters. It also highlights the diverse applications of specific metal ions in enhancing osteogenesis, angiogenesis, antibacterial efficacy, anti-inflammatory and corrosion resistance properties of titanium implants. Furthermore, the review discusses challenges faced and future prospects in this promising area of research. In conclusion, the synergistic approach of micro-arc oxidation and metal ion doping demonstrates substantial promise in advancing the effectiveness of biomedical titanium and its alloys, promising improved outcomes in medical implant applications.

## 1 Introduction

Ever since Professor Brånemark’s discovery of the exceptional biocompatibility of pure titanium and his proposal of the osseointegration theory, titanium and its alloys have found wide application as materials for repairing and replacing hard tissues in fields such as oral and orthopedic surgery. Their advantageous mechanical properties, corrosion resistance, and superb biocompatibility have made them a staple in the production of bone implants, including joint prostheses, fracture fixation devices, and dental implants (Zhang et al., 2013).

Nonetheless, the absence of bioactivity and osteoinductive properties on the surface of titanium implants can impact their osseointegration. In recent years, the fabrication of nano/micron coatings on the surface of titanium and its alloys has gained recognition as a means to enhance their biological attributes and boost their biological activity (van Hengel et al., 2021; Williams, 2022; Kuroda et al., 2023). Various techniques can be employed for creating these coatings, including hydrothermal treatment, physical vapor deposition, chemical vapor deposition, sol-gel methods, plasma immersion ion implantation, selective laser melting, plasma spraying, magnetron sputtering, and micro-arc oxidation (MAO) (Gu et al., 2004; Velasco-Ortega et al., 2010; Li et al., 2014; Wang et al., 2014; Kaluderovic et al., 2015; Zhang et al., 2015; Zhang Y. et al., 2018; Kaya et al., 2018; Kondyurin et al., 2018; Nichol et al., 2021; Bhatti et al., 2022; Sun Z. et al., 2022). However, these coating methods come with certain limitations (refer to Supplementary Table S1), such as reduced fatigue strength, degradation, and poor adhesion to the metal substrate. In contrast to the techniques mentioned above, MAO stands as an *in-situ* surface modification method that produces bioactive coatings on metal surfaces. By manipulating the electrolyte and corresponding voltage or current parameters, a coarse and securely adhered oxide ceramic layer is formed on the surface of titanium and its alloys through the transient high-temperature effects of arc discharge. MAO has been extensively explored for the purpose of enhancing the bioactivity of titanium implants. It has the potential to enhance the surface roughness and energy of the samples, thereby facilitating cell adhesion onto the surfaces of titanium and its alloys (Xiu et al., 2016).

## 2 Manuscript formatting

### 2.1 Current research status of metal ion doping via micro-arc oxidation on the surface of titanium and its alloys

**FIGURE 1:**
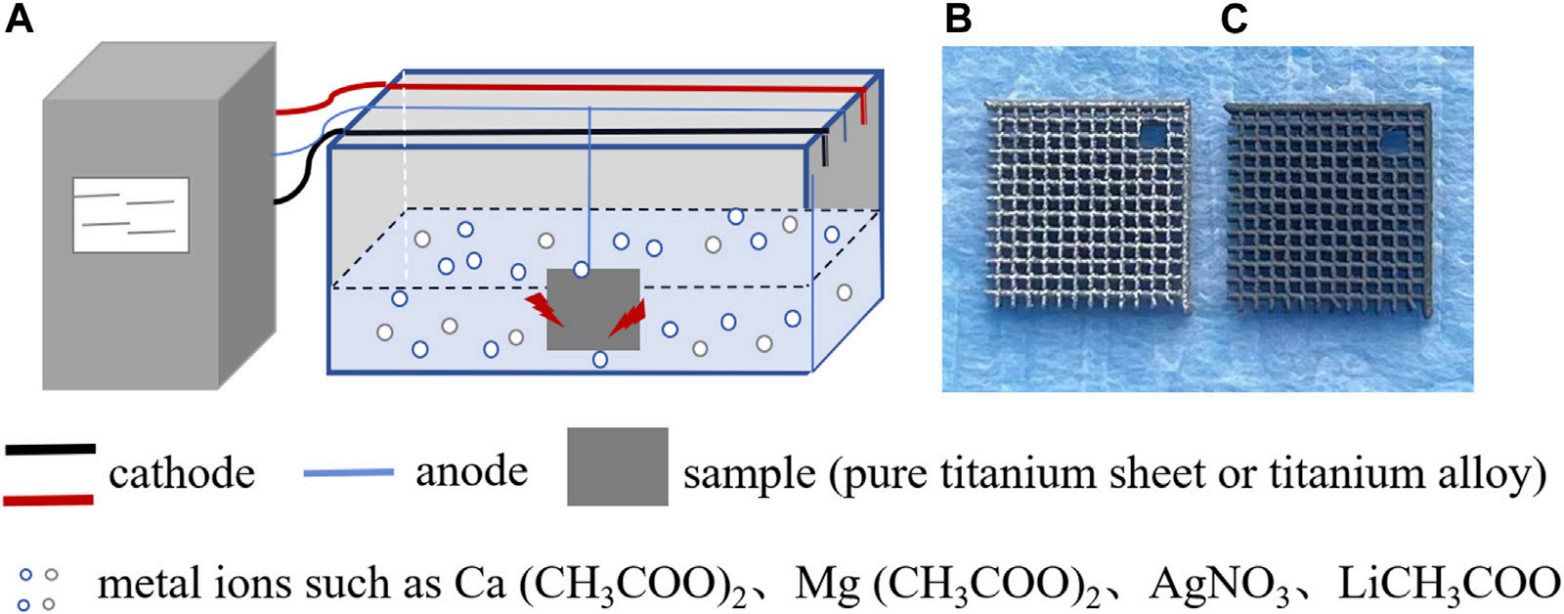
Schematic diagram illustrating the addition of metal ions to the surface coating of titanium and its alloys using micro-arc oxidation technology. **(A)** Micro-arc oxidation process: electrolytic tanks are placed in the electrolyte containing metal ions, and a titanium sheet or titanium alloy is connected to the anode. By adjusting the voltage, current, and time parameters, a stable coating of metal ions can be prepared on the surface of the titanium sheet or titanium alloy. **(B)** Top view of the surface of titanium alloy without micro-arc oxidation treatment. **(C)** Top view of the surface of titanium alloy prepared by micro-arc oxidation treatment with a metal ion coating.

In the medical industry, commercially pure titanium (grades II-IV) and titanium alloys (such as Ti-6Al-4V, Ti-40Nb, Ti-13Nb-13Zr, Ti-35Nb-2Ta-3Zr, and Ti-3Zr-2Sn-3Mo-25Nb) are the most commonly employed materials for implant applications. Micro-arc oxidation (MAO), also referred to as plasma electrolytic oxidation (PEO), anodic spark deposition (ASD), and plasma chemical oxidation (PCO), represents a surface modification technique capable of forming tailored oxide layers on the surface of titanium and its alloys by precisely controlling the composition of the electrolyte. This technique offers notable advantages, including the creation of nano-scale porous structures and the facilitated long-term controlled release of metal ions. As depicted in Figure 1, MAO finds extensive use in the surface modification of titanium and its alloys.

**FIGURE 2:**
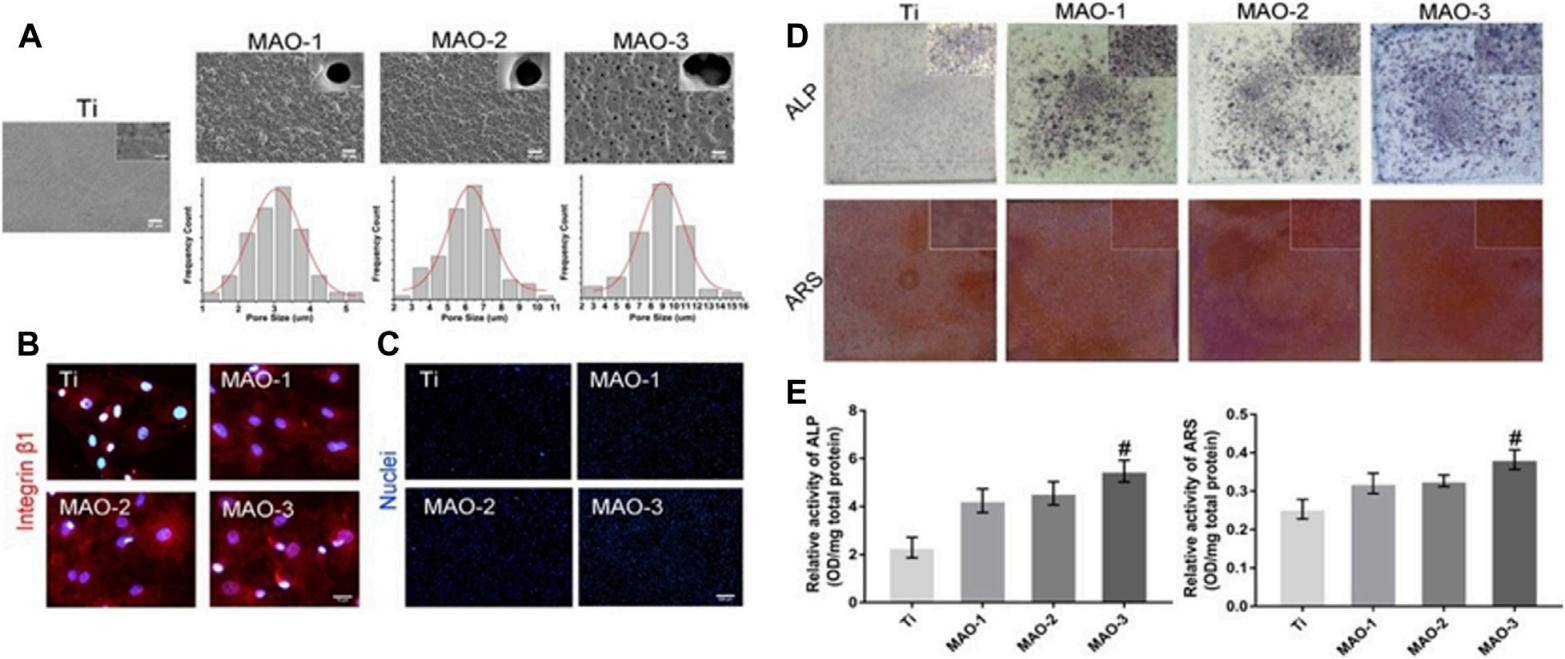
Microporous coatings can be prepared inside and on the surface of titanium scaffolds by adding Ca/P through micro-arc oxidation technology. These microporous coatings promote cell adhesion and osteogenic differentiation. **(A)** SEM images depicting coatings with different pore sizes prepared by micro-arc oxidation. **(B)** Immunofluorescence staining results of Integrinβ1 in BMSCs grafted cells at 4 h **(C)** BMSCs cell count at 4 h post-grafting. **(D)** ALP/ARS staining of BMSCs on coated surfaces with various pore sizes for 7 days. **(E)** Semi-quantitative ALP/ARS detection results of BMSCs on coatings with different pore sizes. The findings demonstrate that the Ca/P coating, produced by micro-arc oxidation on the surface of titanium, exhibits excellent biocompatibility and promotes the adhesion and osteogenic differentiation of bone marrow mesenchymal stem cells.

It is widely recognized that natural bone comprises a variety of elements, including calcium (Ca), magnesium (Mg), strontium (Sr), zinc (Zn), copper (Cu), and others, which play vital roles in bone formation and other biological processes. In the realm of bone tissue engineering, the incorporation of metallic elements can compensate for the osteoinductivity limitations of existing inorganic scaffold materials, making it a potent modification strategy. Towards the close of the last century, the Micro-Arc Oxidation (MAO) technique found application in modifying the surfaces of dental implants. This approach enhances coating biological activity and fosters bone integration by introducing metal ions onto the titanium surface (Li Z. et al., 2019). Our earlier research corroborated these findings (Zhou et al., 2019) (refer to Figure 2). Throughout the process of bone integration between titanium implants and bone tissue, the interaction between these coatings and the titanium substrate raises notable considerations (Velasco-Ortega et al., 2010). Coatings produced via MAO, with the inclusion of doped metal ions, augment the contact area between titanium implants and host tissues, significantly boosting the surface’s biological activity for both titanium and its alloys. This enhancement accelerates the process of bone integration (Bhatti et al., 2022). Multiple studies have demonstrated that the integration of ion substances (such as Ca, Mg, Sr, Zn, Cu, etc. (Xiu et al., 2016; He et al., 2020; Zhang R. et al., 2021; Shen et al., 2022; Yu et al., 2023)) through MAO can refine the chemical characteristics of natural titanium dioxide coatings. By modifying surface energy and chemical morphology, these substances improve early bone implant responses and ensure effective implant surface modifications, consequently enhancing surface bioactivity. The distinct advantages of various metal elements lend themselves to the proposition of adding diverse metal elements to the surfaces of titanium and its alloys. This, in turn, can stimulate osteogenesis, induce blood vessel formation, exhibit antibacterial and anti-inflammatory properties, collectively bolstering implant osseointegration.

While there have been numerous experiments reported on the utilization of MAO to introduce metal elements for modifying the biological activity of titanium implants, and some advancements have been achieved in investigating the biological properties of various metal elements under different MAO preparation parameters, there exists a dearth of comprehensive summaries and reviews in this domain. This paper presents a comprehensive overview of research progress concerning the incorporation of diverse metal ions into surface coatings of titanium materials using MAO. It delineates and compares the osteogenic, angiogenic, antibacterial, and anti-inflammatory effects of distinct metal elements in surface coatings of titanium and its alloys. The ultimate goal is to offer insights for the fabrication of titanium implants exhibiting optimal biological activity.

### 2.2 Commonly employed metal ions for fabricating surface coatings on titanium and its alloys

#### 2.2.1 Calcium ion (Ca^2+^)

Calcium predominantly accumulates in bones and teeth in the form of hydroxyapatite, serving as a fundamental constituent of bone tissue. Hydroxyapatite is also a crucial element in commercial materials used for bone substitutes, scaffolds, and coatings (Bayat et al., 2017). Ca^2+^ functions as a pivotal intracellular signaling ion, orchestrating biological processes by regulating the transcription of numerous genes and transcription factors in all cells within the human body (Karadjian et al., 2019). It not only initiates signal during events such as cell proliferation, mitosis, and differentiation but also governs the cell cycle and enhances cell adhesion. Throughout tissue remodeling, local fluctuations in Ca^2+^ concentration can impact cellular behavior. Studies have demonstrated that lower concentrations of Ca^2+^ facilitate the migration of human osteoblasts, while higher concentrations promote their differentiation, thereby facilitating cell spreading and focal adhesion contacts (Lei et al., 2018). A significant body of research has substantiated that Ca^2+^ can stimulate osteogenesis. The release of Ca^2+^ from biomaterial surfaces can activate calcium ion channel transport proteins and one or more downstream signaling pathways, including the MAPK, cAMP-PKA, and PI3K-AKT pathways, thereby fostering osteoblast differentiation (Liu et al., 2014). It has also been suggested that Ca^2+^ is involved in regulating osteoblast differentiation through the activation of the Wnt/β-catenin signaling pathway, inducing heightened expression of osteogenic differentiation markers in mesenchymal stem cells, and consequently fostering bone formation (Bolander et al., 2016). Thus, the introduction of Ca^2+^ into coatings applied to the surface of titanium and its alloys can modify their surface chemistry. The integration of Ca/P ions into titanium implants enhances their interaction with surrounding bone, directly influencing the expression of osteogenic-related genes and the entire process of bone integration, thereby augmenting bone cell responses to the implants (Li et al., 2004). Such surfaces containing calcium can regulate the cell cycle of bone cells, encouraging osteoblast adhesion, proliferation, and differentiation, thus having the potential to enhance bone integration. Calcium salts exhibit good solubility (under acidic conditions) and can dissolve in water without precipitating when in contact with various electrolytes, enabling calcium to be directly incorporated into titanium implant surface coatings through a single MAO technique (Li et al., 2004; Ribeiro et al., 2015; Huang et al., 2018c; Rokosz et al., 2018; Li Y. et al., 2020; Kuroda et al., 2023), as illustrated in Supplementary Table S2.

#### 2.2.2 Strontium ion (Sr^2+^)

**FIGURE 3:**
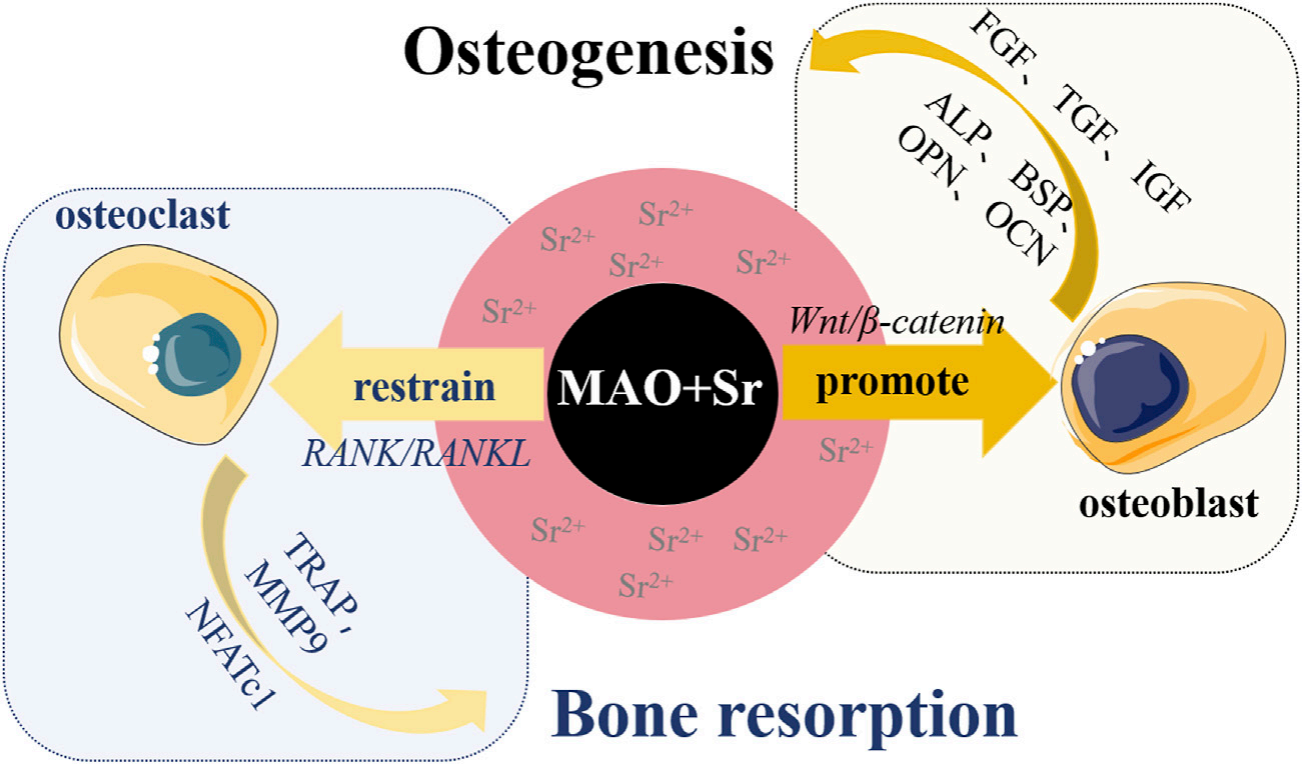
Schematic diagram illustrating the promotion of bone formation by incorporating Sr^2+^ on the surface of titanium-based materials. The incorporation of Sr^2+^ has a dual effect: firstly, it promotes the adhesion and proliferation of osteoblasts by activating the classical Wnt/β-catenin signaling pathway. This activation increases the expression of proteins and growth factors such as alkaline phosphatase (ALP), bone sialoprotein (BSP), osteocalcin (OCN), osteopontin (OPN), insulin-like growth factor (IGF), transforming growth factor (TGF), fibroblast growth factor (FGF), stimulating osteogenic differentiation and mineralization of osteoblasts, ultimately promoting bone formation. Secondly, Sr^2+^ regulates osteoclast differentiation through the RANK/RANKL axis, inhibiting the expression of osteoclast-associated genes like tartrate-resistant acid phosphatase (TRAP), matrix metalloproteinase (MMP9), and activated T-nuclear factor c1 (NFATc1), thereby suppressing bone resorption driven by osteoclasts.

Strontium is a vital trace element present within the human skeletal system, belonging to the alkaline earth metal group alongside calcium. It holds close ties to bone metabolism and can substitute for calcium to create strontium hydroxyapatite, a compound that can coexist with calcium in biomaterials. Extensive research has indicated that strontium exerts a dual impact on bone cells, promoting osteoblast differentiation while inhibiting osteoclast activity (Finke et al., 2007; Pemmer et al., 2013). The recently developed drug strontium ranelate has emerged as a highly effective treatment for osteoporosis. Furthermore, stable strontium exhibits low toxicity, enabling its long-term administration at high doses (Dahl et al., 2001). Sr^2+^ significantly influences the equilibrium between osteoblasts and osteoclasts and aids in bone mineralization (see Figure 3). In tissues rich in osteoblasts, Sr^2+^ activates the classical Wnt/β-catenin signaling pathway, thereby boosting the synthesis of collagen and non-collagen proteins. This, in turn, stimulates osteoblast proliferation and elevates the expression of alkaline phosphatase (ALP) and bone sialoprotein (BSP), facilitating differentiation and mineralization (Yang F. et al., 2011; Cai et al., 2016). Consequently, Sr^2+^ contributes to osteoblast-mediated bone formation (Chen et al., 2021). Conversely, Sr^2+^ intervenes in the NF-κB pathway to regulate osteoclast differentiation through the RANK/RANKL axis (Zhu et al., 2016; Hu et al., 2017). This interference suppresses the expression of mRNA and genes linked to osteoclasts (Mi et al., 2017), thereby restraining osteoclast-driven bone resorption (Wu et al., 2022; Wu et al., 2023).

Materials that incorporate strontium, such as strontium-doped titanium implants, bioactive glasses, bioactive ceramic hydroxyapatite/silicate, and strontium-doped calcium polyphosphate, have exhibited exceptional biocompatibility and osteogenic properties (Park et al., 2012; Yan et al., 2013; Zhang et al., 2016a; Sato et al., 2016; Shen et al., 2022). Nevertheless, animal experiments in this realm are still in their preliminary stages, and the precise mechanisms underlying Sr^2+^'s effects on precursor cells remain elusive, necessitating further comprehensive research. Most strontium salts display good solubility, with 2%–25% of strontium being able to be integrated into the surface coatings of titanium implants using a single MAO technique (Fonseca and Brandi, 2010; Yan et al., 2013; Sato et al., 2016; Zhao et al., 2019b; Wang Y. R. et al., 2023), as outlined in Supplementary Table S2.

#### 2.2.3 Zinc ion (Zn^2+^)

Zinc, an essential trace element, plays a pivotal role in cellular development, DNA synthesis, enzyme activity, and biomineralization. It is integral to diverse metabolic and cellular signaling pathways, contributing to normal growth, immune function, and neural development (Storrie and Stupp, 2005). As a metal ion, zinc exhibits osteogenic and antibacterial properties. Adequate zinc levels on biomaterial surfaces have demonstrated the capacity to stimulate bone cells, initiating a cascade of *in vivo* responses including adhesion, diffusion, proliferation, osteogenic differentiation, and bone formation and mineralization. Zn^2+^ elevates ALP activity and fosters the expression of osteogenic-related genes, such as Runx2, thereby enhancing osteoblast proliferation and the extension of pseudopodia (Thian et al., 2013; Shen et al., 2014; Zhang R. et al., 2018). Furthermore, cellular and molecular evidence suggests that Zn^2+^ acts as a vital cofactor for ALP and collagenase. Supplementation with Zn^2+^ or its incorporation into biomaterials upregulates the expression of osteogenic-related genes (e.g., ALP, collagen type I (COL-I), osteocalcin (OCN), and osteopontin (OPN)), thereby inducing osteoblast differentiation, and subsequently enhancing collagen secretion and calcium deposition, leading to the formation of bone nodules (Hadley et al., 2010; Yusa et al., 2011).

Research has indicated that zinc’s antibacterial properties are stable, long-lasting, and less susceptible to resistance compared to antibiotics (Zackular et al., 2016). Zn^2+^ can disrupt bacterial cell membrane integrity, trigger protein denaturation, and cause cellular content leakage, thus exerting antibacterial effects (Jin et al., 2014). Additionally, Zn^2+^ can impede bacterial cell wall synthesis, hindering the growth and reproduction of bacteria (Ahmed et al., 2019). Some studies have also highlighted zinc’s potential to enhance macrophage phagocytic activity and the release of inflammatory cytokines in polymorphonuclear leukocytes (Wang et al., 2017). Despite its established roles in bone metabolism, the clinical viability of zinc-containing biomaterials hinges on several factors, particularly concerns regarding zinc content and release kinetics (Qiao et al., 2014).

Zinc-enriched titanium implants have the potential to boost bone formation by facilitating the osteogenic differentiation of bone marrow mesenchymal stem cells (BMSCs) while concurrently deterring bacterial adhesion and growth, thus mitigating inflammation surrounding the implants (Hu et al., 2012; Ahmed et al., 2019). The integration of zinc onto titanium implant surfaces represents a trend in cationic surface modification, with the release of Zn^2+^ significantly influencing bone integration. Zinc salts, such as zinc acetate, possess good water solubility, and a single MAO technique can be employed to immobilize zinc onto the surface coatings of titanium implants (Hu et al., 2012; Du et al., 2018; Wang et al., 2018; Zhao et al., 2019b; Komarova et al., 2020; Sun H. et al., 2022), as illustrated in Supplementary Table S2.

#### 2.2.4 Magnesium ion (Mg^2+^)

Magnesium, an essential element crucial for bone health, ranks as the second most abundant divalent cation within cells, exerting a vital role in regulating diverse cellular functions. Roughly 60% of the body’s magnesium is stored in the bone matrix, where it governs the transport of calcium and potassium ions, thereby maintaining structural integrity and functional equilibrium. Additionally, magnesium acts as a cofactor for enzyme activation and inhibition, influencing cellular processes such as cell cycle control, proliferation, and differentiation. Hence, magnesium deficiency can precipitate osteoporosis, making Mg^2+^ supplementation particularly beneficial for osteoporosis patients (Zhang Y. et al., 2016). Mg^2+^ exercises its influence on bone mineral and matrix metabolism via bone metabolism-associated hormones, growth factors, signaling pathway elements, as well as by direct interaction with bone tissue itself (Yan et al., 2019). The influence of magnesium on bone metabolism chiefly manifests in the activation of osteoblasts, fostering the proliferation, differentiation, and adhesion of osteoprogenitor cells. However, excessive Mg^2+^ levels can hinder these processes and extracellular matrix mineralization by competing with Ca^2+^ for calcium ion channels, ultimately promoting bone development.

Studies have indicated that Mg^2+^ enhances the initial biological response surrounding implants and augments the biomechanical strength of bone integration (Reis et al., 2020). Furthermore, as a biodegradable element, magnesium has attracted extensive research attention for its incorporation into biomaterials to facilitate stem cell differentiation into osteoblasts and amplify bone formation (Sun et al., 2013; Li X. et al., 2018; Nabiyouni et al., 2018; Zhao Z. et al., 2021). Capitalizing on the biological attributes of magnesium ions, surface modification techniques utilizing magnesium-containing cations have emerged as a focal point in biomedical exploration. In the realm of bone regeneration, research has unveiled the benefits of introducing magnesium to titanium implants, fostering improved bone integration (Wang et al., 2014; Li X. et al., 2020; Zhang R. et al., 2021), and integrating Mg^2+^ onto nano-porous titanium surfaces to enhance osteoblast maturation (Sul et al., 2005; Wang et al., 2014; Zhao et al., 2019a). The optimal concentration of Mg^2+^ can activate downstream target proteins by modulating Akt expression, thus influencing osteoblast proliferation and differentiation (McGonnell et al., 2012). Moreover, Mg^2+^ has been found to polarize macrophages toward an M2 phenotype, curbing macrophage inflammatory responses *in vitro* (Li X. et al., 2018). Elevated Mg^2+^ concentrations are linked to reduced production of reactive oxygen species (ROS) and nitric oxide (NO) in immune cells (Son et al., 2007). Additional investigations have demonstrated Mg^2+'^s capability to foster vascular regeneration by activating the e-NOS signaling pathway (Vinten-Johansen et al., 2005; de Baaij et al., 2015).

Magnesium salts, such as magnesium acetate, exhibit favorable water solubility and can be integrated into the surface coatings of titanium implants through the single technique of micro-arc oxidation (MAO) (Li X. et al., 2018; Li X. et al., 2020; Zhang R. et al., 2021), as illustrated in Supplementary Table S2.

#### 2.2.5 Copper ion (Cu^2+^)

Copper is an essential trace element within the human body, renowned for its involvement in bone metabolism and antimicrobial properties (Hoppe et al., 2011; Burghardt et al., 2015). *In vitro* investigations have demonstrated that external Cu^2+^ stimulates ALP activity, augments the synthesis of COL-I, OCN, and OPN, enhances *in vitro* mineralization, and encourages osteogenic differentiation of mesenchymal stem cells (MSCs) within an osteogenic culture medium (Burghardt et al., 2015). Studies have further indicated that the integration of Cu^2+^ into biomaterial surfaces can induce the generation of ROS, leading to potential damage to lipids, proteins, membranes, and DNA (Borkow and Gabbay, 2005; Nandakumar et al., 2011; Huang et al., 2018a). Furthermore, surfaces containing copper have been shown to enhance macrophage-mediated bacterial phagocytosis and augment bacterial eradication, suggesting an improved bactericidal capacity of macrophages in the presence of copper (Huang et al., 2018b; Huang et al., 2019). Another investigation exhibited that copper nanoparticle coatings, applied to biomaterial surfaces, manifest remarkable antibacterial effects. Even at low concentrations, these copper nanoparticles exhibit favorable biocompatibility, fostering osteoblast proliferation and adhesion, as well as heightening extracellular matrix mineralization (Zhang X. et al., 2018). Moreover, both low and high concentrations of copper nanoparticles are demonstrated to be non-toxic to endothelial cells and to stimulate the release of nitric oxide and vascular endothelial growth factor (Zhang X. et al., 2018). Numerous studies have corroborated the interaction of Cu^2+^ with various angiogenesis-related growth factors, including vascular endothelial growth factor (VEGF), angiogenin (ANG), and matrix metalloproteinases (MMPs) (Jacobs et al., 2020). The addition of Cu^2+^ to bone regenerative materials can significantly elevate the expression of hypoxia-inducible factor-1α (Hif-1α), consequently upregulating the expression of genes and proteins associated with angiogenesis (Gerard et al., 2010; Wu et al., 2013).

While the optimal concentration of Cu^2+^ within diverse biomaterials has been extensively explored, some literature has indicated that elevated Cu^2+^ concentrations might induce cytotoxic effects and inhibit cellular activity (Zhang et al., 2016b). Hence, it is imperative to ensure the safety of copper-containing biomaterials. An optimal strategy to achieve this safety entails maximizing osteogenic rates both *in vitro* and *in vivo* while minimizing the copper content. Copper salts, such as copper sulfate, possess notable water solubility under acidic conditions, allowing the incorporation of copper ions (Huang et al., 2018a; Huang et al., 2019; Shimabukuro et al., 2020) or copper nanoparticles (Zhang X. et al., 2018) into surface coatings of titanium implants through the utilization of the single MAO technique, as illustrated in Supplementary Table S2.

#### 2.2.6 Cobalt ion (Co^2+^)

Cobalt is an essential trace element and functions as a cofactor for numerous metalloproteins within the human body (Fraga, 2005). Co^2+^ has been demonstrated to modulate the expression of various genes associated with cytokine, chemokine, and regulatory molecule production, which play crucial roles in osteoblast functionality. In the realm of osteogenesis, mesoporous bioactive silicate glass scaffolds containing low concentrations of Co^2+^ have been observed to amplify the osteogenic response of bone marrow stromal cells (Wu et al., 2012). Furthermore, Co^2+^ can stabilize Hif-1α and subsequently activate downstream target genes like VEGF, making it a common choice for *in vitro* simulation of hypoxic environments (Wu et al., 2012). Nevertheless, excessive doses or prolonged exposure to Co^2+^ can impede cell growth, provoke cellular damage, and even induce apoptosis, potentially stemming from heightened oxidative stress (Lee et al., 2013). Research suggests that this cellular impairment is instigated by the generation of reactive oxygen species through the activation of Erk1/2 and NF-κB signaling pathways (Yang C. et al., 2011). Notably, elevated Co^2+^ levels have the potential to induce cytotoxic effects, underscoring the critical importance of ascertaining optimal and safe dosages when integrating it into implant surfaces (Zhou and Zhao, 2016b).

Extensive investigations have illustrated that cobalt can be incorporated into titanium alloy scaffolds through the utilization of MAO techniques. *In vitro* studies employing mesenchymal stem cell (MSC) culture models have indicated that titanium alloy scaffolds containing cobalt exhibit favorable cell compatibility, heightened angiogenesis, and osteogenic attributess (Wu et al., 2012; Perez et al., 2015; Zhou and Zhao, 2016a). Cobalt salts (e.g., cobalt acetate) possess good water solubility and can be integrated into the surface coatings of titanium implants using a singular MAO approach (Zhou and Zhao, 2016a; Zhou and Zhao, 2016b; Zhao Q. M. et al., 2021), as outlined in Supplementary Table S2.

#### 2.2.7 Silver ion (Ag^+^)

Silver is one of the trace elements present in human tissues and has been utilized for thousands of years as a natural antibacterial material due to its extensive antibacterial spectrum and reduced propensity for generating resistance. In recent times, silver nanoparticles (AgNPs) have garnered significant interest as a classical antibacterial agent in the realm of antibacterial activity (Zhang Y. Y. et al., 2021; Wang H. et al., 2023; Wang Y. R. et al., 2023; Maj et al., 2023). The appeal of AgNPs can be attributed to various factors. Firstly, silver particles possess a substantial surface area-to-volume ratio and robust penetrability. Secondly, AgNPs exhibit remarkable bactericidal properties, ensuring prolonged resilience against bacteria. Additionally, they boast high-temperature stability and low volatility, yielding enduring antibacterial effects. The antibacterial mechanisms of silver are widely acknowledged through two modalities: contact-based eradication and ion-mediated eradication. AgNPs can attach to bacterial cell walls, inducing membrane damage that leads to leakage of intracellular contents and bacterial demise. The release of Ag^+^ ions from AgNPs also assumes a pivotal role in displaying antibacterial efficacy. The antibacterial potential of Ag^+^ ions has been substantiated (Calabrese et al., 2021); however, it may impose adverse effects on cells, underscoring the significance of controlled Ag^+^ release to mitigate unwanted outcomes.

Silver has additionally exhibited anti-inflammatory attributes and the capacity to induce the differentiation of stem cells into osteoblasts. Consequently, the incorporation of Ag^+^ ions onto the surface of titanium implants using MAO techniques can augment the initial adherence of BMSCs. Ag^+^ ions collaborate with the MAPK/ERK signaling cascade to activate osteogenic markers, enhance ALP activity and mineralization levels, and upregulate the expression of osteogenic genes, culminating in enhanced bone integration (Shimabukuro et al., 2019). Hence, incorporating silver into the surface of titanium and its alloys can enhance the antibacterial, anti-inflammatory, and osteogenic properties of titanium implants. Silver salts (such as silver nitrate) exhibit good water solubility and can be incorporated into the surface coatings of titanium implants using a single MAO technique. Thus, the integration of silver onto the surfaces of titanium and its alloys can heighten the antibacterial, anti-inflammatory, and osteogenic properties of titanium implants. Silver salts (e.g., silver nitrate), with favorable water solubility, can be seamlessly incorporated into the surface coatings of titanium implants through a singular MAO technique (Jia et al., 2016; Shimabukuro et al., 2019; Zhang L. et al., 2020; Zhang Y. Y. et al., 2021; Sun H. et al., 2022; Maj et al., 2023), as depicted in Supplementary Table S2.

#### 2.2.8 Other metal cations

There are several other metal cations that can be incorporated onto the surface of titanium implants using MAO technology, including manganese (Mn), iron (Fe), lithium (Li), vanadium (V), aluminum (Al), gold (Au), and others (refer to Supplementary Table S2). Among these, the absence of manganese (Mn) may lead to issues such as delayed bone formation and bone deformities. Coatings containing manganese, produced through MAO technology on the surface of titanium implants, exhibit remarkable corrosion resistance and can release Mn^2+^ over an extended duration, thereby exerting inhibitory effects on *Escherichia coli* and *Pseudomonas aeruginosa*. Concerning osteogenesis, Mn-doped titanium implants have demonstrated significant contributions to bone formation (Kang et al., 2018). Manganese can enhance osteoblast differentiation by influencing the parathyroid hormone signaling pathway, regulating bone mineral density, and augmenting overall bone formation (Zhang X. et al., 2020). However, excessive levels of manganese can have toxic effects on osteoblasts. Regarding iron (Fe), studies have demonstrated that Fe^2+^ incorporated within titanium dioxide coatings can chemically disrupt cell membranes and notably enhance the antibacterial activity of MC3T3-E1 cells (Tian et al., 2014). Increasing Fe^2+^ concentration can enhance fibroblast responses, encompassing proliferation, phenotype, and extracellular collagen secretion (Li K. et al., 2019), while also promoting osteoblast proliferation, expression of osteogenic genes, and extracellular matrix mineralization (Li K. et al., 2018). Recent research concerning lithium (Li) has indicated that employing MAO technology can generate titanium scaffolds with lithium-containing nano-porous coatings. In both *in vitro* and *in vivo* experiments, Li^+^ has shown to significantly enhance the biocompatibility and osteogenic potential of bone repair materials. Additionally, it has been demonstrated to stimulate the expression of ALP and osteogenic genes in osteoblasts through activation of the classical Wnt/β-catenin signaling pathway (Liu et al., 2018).

## 3 Enhancing the biological performance of titanium and its alloy surfaces through metal cation coatings conclusion

During the process of osseointegration between titanium implants and the surrounding bone tissue, essential factors encompass antimicrobial, anti-inflammatory, osteoinductive, and angiogenic effects. Employing MAO technology facilitates the creation of coatings enriched with active metal elements on the surfaces of titanium and its alloys, thereby enhancing their biological activity. The incorporation of exogenous metal cations with diverse biological properties onto the surfaces of titanium implants can ameliorate osseointegration, bolster material corrosion resistance, and confer controlled or amplified antimicrobial, anti-inflammatory, osteoinductive, and angiogenic characteristics in accordance with specific requirements. Nevertheless, many of the modifications introducing metal cations to implant surfaces are confined to elements possessing outstanding properties. Thus, investigating strategies that integrate assorted metal cations onto titanium implant surfaces and harness their optimal synergistic effects constitutes a burgeoning area of research.

### 3.1 Enhancing osteoinductive performance of Titanium and its alloy surfaces through coatings with metal cations

Enhancing osteoinductivity is a pivotal objective in the context of titanium implants. The enduring stability of titanium implants hinges on the firm osseointegration established at the bone/implant interface. To ensure successful osseointegration, it is imperative that titanium implants facilitate the preferential differentiation of bone marrow mesenchymal stem cells into osteoblasts. Consequently, the formulation of coatings featuring osteoinductive metallic elements on the surface of titanium implants stands as an efficacious strategy to augment their osteogenic biological performance. Extensive investigation has been undertaken concerning metal elements with inherent osteoinductive properties such as Calcium (Ca), Strontium (Sr), Zinc (Zn), Magnesium (Mg), and more, specifically at the cellular level, corroborating their efficacy in osteogenic applications (refer to Supplementary Table S3). As an illustration, considering Sr (as demonstrated in Figure 3), its incorporation into implant surfaces has been proven to markedly accelerate early bone integration, both under normal conditions and in cases of osteoporosis (Shen et al., 2022; Yan et al., 2022). Nevertheless, the precise molecular mechanisms through which osteoinductive metal elements foster bone formation remain partially elucidated. Thus, a comprehensive synthesis of research pertaining to the incorporation of osteoinductive metal cations into coatings on titanium implant surfaces, elucidation of their optimal and safe ion concentration ranges, and a deeper understanding of their biological impacts and mechanisms underpinning bone formation promotion are indispensable (see Supplementary Table S3).

### 3.2 Enhancing vascularization performance of titanium and its alloy surfaces through metal cation coatings

**FIGURE 4:**
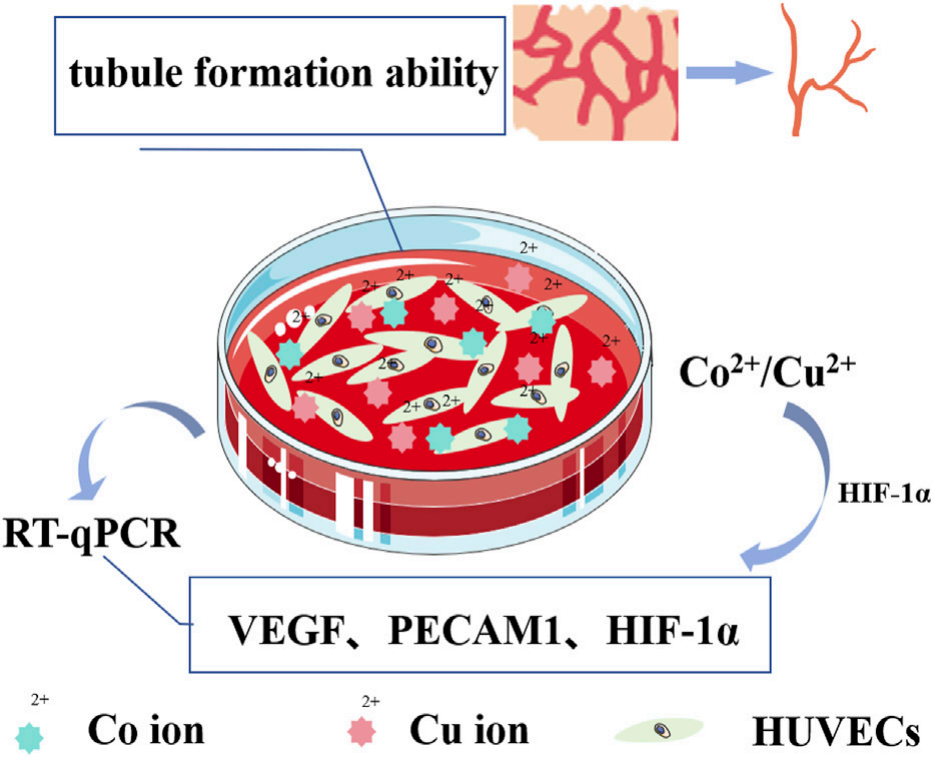
Schematic diagram illustrating angiogenesis induced by Cu^2+^/Co^2+^. Cu^2+^ enhances the angiogenic response by upregulating the expression of hypoxia-inducing-factor-1α (HIF-1α), thereby initiating the transcription of vascular endothelial growth factor (VEGF). Simultaneously, Co^2+^, acting as a prolyl hydroxylation inhibitor, inhibits the degradation of HIF-1α, maintaining its stability. The expression of HIF-1α and VEGF is induced in a dose-dependent manner, and the co-culture of HUVEC with Cu^2+^/Co^2+^ culture medium *in vitro* increases the expression of VEGF, platelet endothelial cell adhesion molecule (PECAM1), and HIF-1α-related genes in HUVEC. This enhancement significantly augments the ability of HUVEC to form tubular structures.

The inadequate vascularization activity observed in titanium implants contributes to suboptimal osseointegration. Vascular formation plays a pivotal role in the initial stages of bone integration post-implantation, as well as in maintaining bone equilibrium. Consequently, promoting vascularization represents a vital area for enhancement in titanium implants. Vascularization serves not only to supply nutrients for new bone growth but also to facilitate the migration of bone marrow mesenchymal stem cells to the implant surface, thus aiding in bone formation. Furthermore, well-established vascular networks contribute to the prevention of infections. Studies have substantiated (refer to Supplementary Table S4) that specific metallic elements, such as Zn, Co, Cu, Sr, Li, etc., when co-cultured with cells, exhibit remarkable angiogenic induction properties. Particularly noteworthy is the robust angiogenic induction exhibited by Co^2+^ and Cu^2+^. For example, when CuCl_2_ and CoCl_2_ were co-cultured with human umbilical vein endothelial cells (HUVECs), it was demonstrated that higher concentrations of Cu^2+^ during the initial phase, followed by lower concentrations of Cu^2+^ in later stages, fostered enhanced vascularization responses (as depicted in Figure 4). In contrast, elevated concentrations of Co^2+^ bolstered the expression of angiogenic genes and the capacity to form tubular structures rich in bioactive molecules (Bosch-Rue et al., 2021). However, upon perusal of pertinent literature, it becomes apparent that studies dedicated to metal cations possessing angiogenic properties remain relatively scarce. Even more sparse are investigations exploring the incorporation of angiogenic metal cations onto titanium implants using the MAO technique. Consequently, a concise synthesis of these studies is presented in Supplementary Table S4.

### 3.3 Enhancing antimicrobial performance of titanium and its alloy surfaces through coating with added metal cations

**FIGURE 5:**
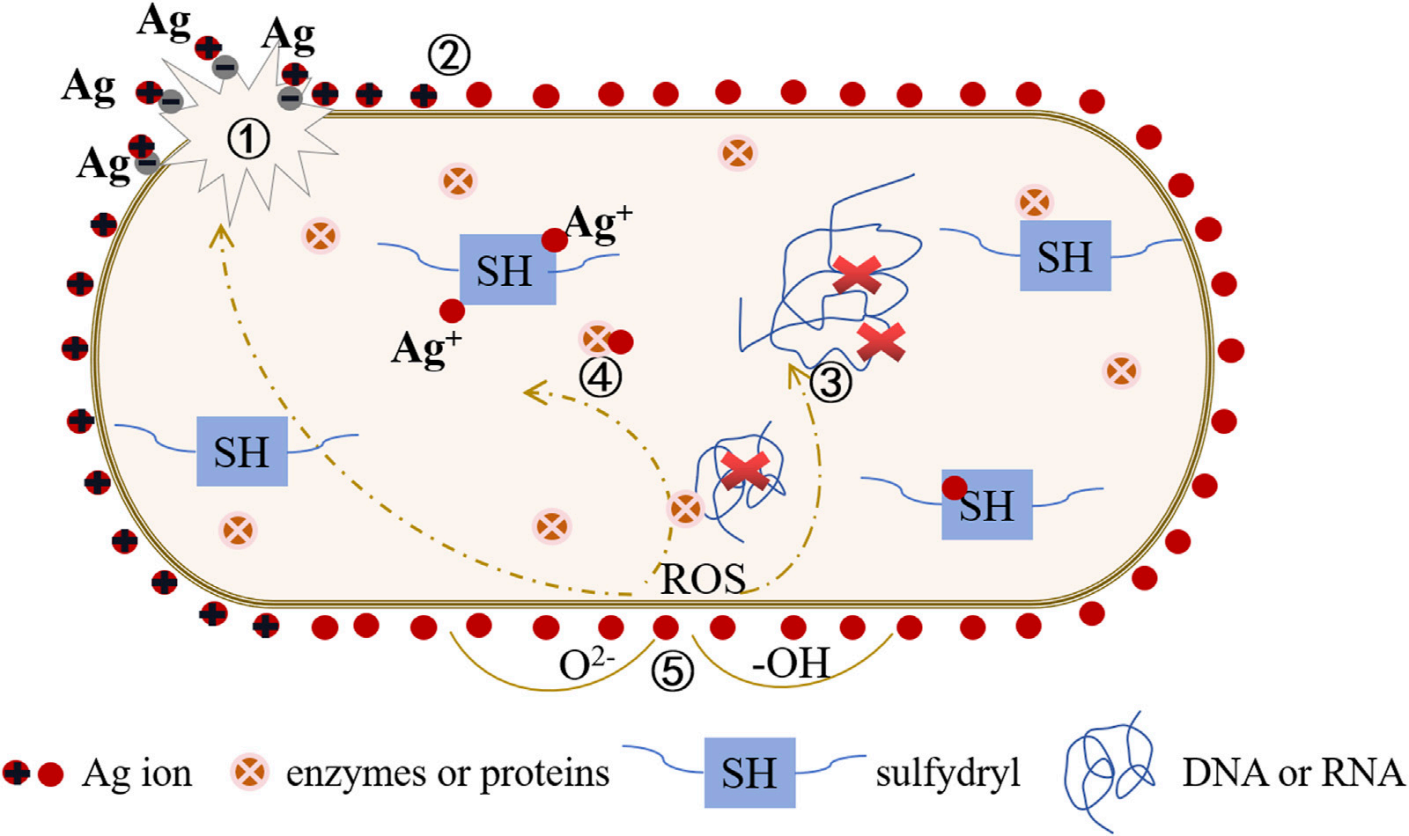
Antibacteriological diagram of the titanium base material surface doped with Ag^+^. ① Interaction with the cell wall: Ag^+^ disrupts the bacterial cell wall, leading to cytoplasmic efflux and ultimately causing bacterial death. ② Electric field adsorption: Ag^+^ accumulates on the surface of the cell membrane, affecting the membrane permeability of bacteria and simultaneously disrupting the electronic transport and material transport systems of bacteria. ③ Interaction with DNA: Ag^+^ generates reactive oxygen radicals (ROS), binds with DNA, replaces hydrogen in the double helix structure of the DNA molecule, causing deformation of the bacterial DNA molecular structure. This inhibits the synthesis of DNA, RNA, and proteins, leading to bacterial inactivation. ④ Interaction with enzymes or proteins: Ag^+^ combines with bacterial groups, such as -SH, causing protein coagulation, disrupting the activity of cell synthesis enzymes, and preventing cell division and proliferation, ultimately leading to bacterial death. ⑤ Catalytic effect: Ag^+^ activates surrounding oxygen, producing hydroxyl radicals (-OH) and reactive oxygen ions (O^2-^), exerting a strong redox effect to hinder microbial cell proliferation and inhibit or kill bacteria.

Titanium implants lack inherent antimicrobial activity, rendering their surfaces susceptible to bacterial adhesion and microbial colonization. This situation can lead to implant-associated infections and heightened implant failure rates. Consequently, various novel antimicrobial strategies have emerged, including photodynamic therapy, sonodynamic therapy, photothermal therapy, chemical dynamic therapy, and metal ion therapy. Among these, metal ion therapy involves the controlled release of metal ions (e.g., Zn, Ag, Cu, etc.) to disrupt the normal physiological functions of bacteria, showcasing remarkable broad-spectrum antimicrobial efficacy. Taking silver (Ag) as an example (refer to Figure 5), Silver ions can react with water molecules to produce a series of harmful free radicals to bacteria, such as hydroxyl radicals (-OH), these free radicals have a strong oxidizing effect, can destroy the biological components of bacterial cells, inhibit the growth of bacteria. At the same time, silver ions will damage the bacterial cell membrane, interfere with bacterial metabolic activities, block bacterial DNA replication and repair, leading to bacterial death.

Currently, the fabrication of antimicrobial metal materials primarily encompasses two approaches. Firstly, there is a focus on developing new alloy materials possessing inherent antimicrobial properties. Secondly, traditional implants, particularly non-degradable metal surfaces, undergo physical and chemical modifications. This section primarily consolidates the chemical modifications of titanium and its alloy surfaces through the MAO technique, introducing metal ions with antimicrobial bioactivity (refer to Supplementary Table S5). This approach amplifies the antimicrobial performance of implants, regulates microbial infections, and enhances implant success rates. However, it is imperative to recognize that the antimicrobial attributes and host cell toxicity of these metal ions are contingent on dosage. Hence, meticulous control over the concentration of metal ions with antimicrobial bioactivity is essential to ensure effective eradication of bacteria on the implant surface while minimizing any deleterious impact on host cells.

### 3.4 Enhancing anti-inflammatory performance of titanium and its alloy surfaces through coatings with metal cations

During the initial stages following implantation, bone biomaterials can incite inevitable inflammatory responses, which are regarded as pivotal determinants of implant outcomes. The initial inflammatory reaction and subsequent bone reconstruction are intricately intertwined, with immune cells, particularly macrophages, playing a pivotal role in tissue repair and regeneration. Proper immune responses can expedite bone formation and bolster osseointegration. Consequently, improving osseointegration necessitates the consideration of bone immunomodulation. Macrophages serve as the vanguard of the innate immune system, exerting a substantial influence on tissue-material interactions via the secretion of chemokines and cytokines. Nevertheless, this factor is frequently overlooked when evaluating the osteogenic potential of bone biomaterials. Upon implantation, titanium implant materials prompt macrophages to colonize the biomaterial surface, thus orchestrating foreign body reactions. Responding to the dynamic local microenvironment, recruited and activated macrophages can polarize into M1 or M2 phenotypes (Zhang H. et al., 2018; Locati et al., 2020). The inherent rapid M1 polarization, followed by a timely M2 anti-inflammatory response, heralds the onset of early bone formation. Furthermore, M2 macrophages secrete cytokines such as Interleukin-10 (IL-10), Transforming growth factor-β(TGF-β), VEGF, and Bone morphogenetic protein 2 (BMP-2), which directly attract osteoblasts and stimulate osteogenic differentiation of bone marrow mesenchymal stem cells (Abaricia et al., 2020; Zhu et al., 2021). Hence, the regulation of macrophage phenotypes bears significance in fostering osseointegration around the implant.

**FIGURE 6:**
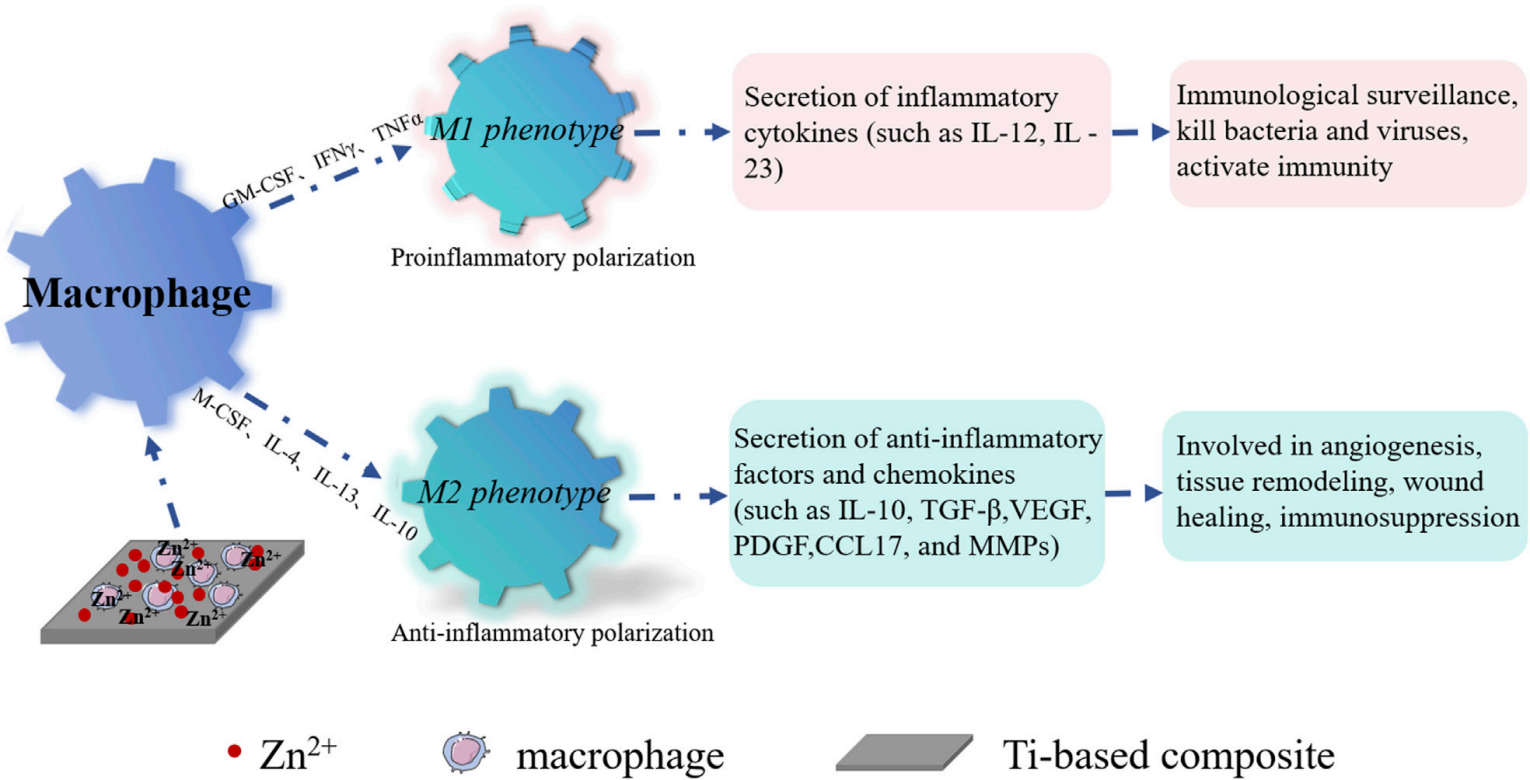
Schematic diagram illustrating anti-inflammation with Zn^2+^ on the surface of the titanium base material. Zn^2+^ interacts with macrophages, induced by granulocyte-macrophage colony-stimulating factor (GM-CSF), interferon-γ (IFN-γ), Tumor Necrosis Factor-α (TNF-α), etc., leading to polarization into M1 macrophages. These M1 macrophages contribute to bacterial and viral elimination, activating immunity. Furthermore, Zn^2+^ interacts with macrophages, induced by macrophage colony-stimulating factor (M-CSF), Interleukin-4 (IL-4), Interleukin-13 (IL-13), Interleukin-4 (IL-10), glucocorticoid, and other factors, ultimately transforming into M2 macrophages. These M2 macrophages play a role in angiogenesis, tissue remodeling, wound healing, and immunosuppression.

Current research demonstrates that incorporating bioactive metal ions, like Sr, Zn, Mg, Cu, etc., into coatings on the surfaces of titanium and its alloys using the MAO technique can significantly amplify the anti-inflammatory potential of titanium implants. Notably, Sr^2+^ within high Sr materials (Sr75% and Sr100%) can propel macrophages to polarize into M2 phenotypes. This is achieved by inhibiting macrophage oxidative stress and inflammatory levels, regulating cell morphology, and catalase (CAT)/superoxide dismutase (SOD) activity. Consequently, M2 macrophages promote the expression of osteogenic cytokines such as TGF-β1 and BMP2, which effectively enhance bone formation and facilitate early osseointegration on titanium implant surfaces (Shen et al., 2022). Coatings of Zn-modified titanium have been found to restrain macrophage adhesion, proliferation, and polarization towards the M2 phenotype, creating an anti-inflammatory microenvironment that fosters healing (see Figure 6). The molecular mechanism underlying Zn’s anti-inflammatory action is attributed to its upregulation of the antioxidant enzyme gene CAT(Song et al., 2010). It is likely that Zn also impacts NF-κB to regulate the pro-inflammatory response^[114]^. Studies indicate that at Zn^2+^ concentrations below or near 80μM, the suppression of pro-inflammatory factor expression is more pronounced, whereas this effect diminishes at concentrations exceeding 80 μM(Sun H. et al., 2022). Additionally, Mg-MAO surfaces can curb inflammatory responses during the pro-inflammatory stage by transitioning macrophages from the M1 phenotype to the M2 phenotype. This involves suppressing M1 markers (CD86, CD11c, and inducible nitric oxide synthase (INOS)) and pro-inflammatory cytokine (Tumor necrosis factor (TNF-α) and Interleukin-1β(IL-1β)) gene expression, while boosting M2 marker CD163 gene expression and curtailing TNF-α release (Li X. et al., 2018). In hybrid coatings of Mg/Zn-MOF74, Zn^2+^ exhibits more potent anti-inflammatory effects on macrophages compared to Mg^2+^(Shen et al., 2019). Furthermore, investigations into the inflammatory response of macrophages on copper-doped surfaces reveal that coatings containing copper on titanium implants can steer macrophages towards the M1 phenotype. This stimulation prompts the release of pro-inflammatory cytokines and suppresses the release of anti-inflammatory cytokines (IL-10 and IL-4) by activating the Cu transport signaling pathway in macrophages (Huang et al., 2018a). More recently, lithium ions have also been incorporated into titanium implants for gradual and sustained release. Research has unveiled that low doses of Li^+^ can bolster macrophage recruitment by modulating the PI3K/AKT signaling pathway. This modulatory effect restrains pro-inflammatory polarization (M1 phenotype) and fosters anti-inflammatory polarization (M2 phenotype), thus mitigating inflammatory reactions around implants (Peng et al., 2021).

### 3.5 Enhancing corrosion resistance performance of titanium and its alloy surfaces through coatings with metal cations

Titanium and its alloys find extensive use in biomedicine and dentistry due to their inherent stability and corrosion resistance in various environments, including body fluids, saliva, and artificial physiological solutions. The formation of a thin passivation film on the surface contributes to this corrosion resistance; however, the presence of microorganisms may disrupt the film, resulting in corrosion. Therefore, evaluating the corrosion resistance of titanium and its alloy surfaces is crucial in studying their biocompatibility. Adding metal ions such as Zn (Lv et al., 2021) or Cu(Xia et al., 2020) to the surface has proven to be an effective method for improving corrosion resistance. Numerous studies (refer to Supplementary Table S6) highlight the positive impact of micro-arc oxidation treatment on the corrosion resistance of titanium and its alloy implants.

## 4 Enhancing surface properties of Titanium implants through the synergy of Micro-Arc oxidation and other techniques for metal cation coating production

Ensuring robust osseointegration heavily relies on the stability of the bone/implant interface (Davies, 2007). The synergy between surface chemistry and micro-topography holds substantial promise for achieving improved stability in the bone/implant connection (Sul, 2003; Davies et al., 2013). Given the complex physiological environment within the human body, coatings applied to titanium and its alloys often exhibit limited stability, potentially impacting the adhesion between titanium implants and surrounding tissues. Therefore, prioritizing the stability of these coatings is crucial. Establishing consistently stable coatings through a single technique presents challenges. To enhance the effectiveness of coatings on titanium and its alloy surfaces, modern fabrication approaches have evolved from single coatings to composite coatings, gradient coatings, nano-gradient coatings, and the simultaneous use of various modification methodologies (Li et al., 2016). Regarding the development of metal-infused coatings on titanium and its alloys, techniques for composite coating preparation have progressively advanced (refer to Supplementary Table S7). These techniques include the integration of micro-arc oxidation with electrochemical or alkali-thermal treatments, ion implantation, electrophoretic deposition, laser cladding, hydrothermal synthesis, and plasma spraying (Han et al., 2011; Tian et al., 2014; Zhang et al., 2016a; Yuan et al., 2017)

## 5 Discussion

The determination of the safety of titanium implant materials is also a critical aspect that should not be overlooked. As evident from the previous summary, substantial work has been done to identify the optimal concentrations of metal elements incorporated into titanium implant materials (Supplementary Table S3–5). Nevertheless, the specific concentrations of metal ions within modified surface coatings of titanium implants and the mechanisms governing the release of metal ions warrant further investigation. Hence, while focusing on the correlation between the concentration of exogenous metal ions on the surface of titanium implants and bone metabolism around the implants, it is imperative to elucidate the molecular mechanisms through which exogenous metal ions impact bone metabolism. By doing so, we can ascertain the optimal concentration and safety thresholds for incorporating exogenous metal cations using MAO technology. This knowledge will facilitate the fabrication of coatings containing multiple elements on titanium implants, offering predictable and controlled biological functionality. These coatings can exhibit enhanced properties such as osteogenesis, angiogenesis, modulation of bone immunity, resistance of corrosion and antimicrobial efficacy, all while accurately modulating the behavior of diverse cell types. Ultimately, these advancements will contribute to the overall improvement of titanium implant performance.

In summary, the incorporation of metal cations during the fabrication of coatings on the surfaces of titanium and its alloys can effectively address the shortcomings in osteoinductivity observed during interactions with seeded cells. This approach proves to be a highly efficient method for modifying inorganic scaffold materials. Many elements possess multifaceted properties, and combining two or more elements can yield a broader spectrum of biological characteristics compared to individual elements. Therefore, future modification techniques involving cations should emphasize the incorporation of multiple metal cations onto the surfaces of titanium implants, enhancing the stimulation of bone metabolism around the implants and overall performance enhancement. Furthermore, future research endeavors may explore the amalgamation of various modification methods or refinements in coating structure and composition, thereby further amplifying the capabilities of the coatings. Although notable strides have been made in incorporating metal cations into coatings via MAO technology, a comprehensive assessment of biocompatibility and safety *in vitro* and *in vivo* remains limited. However, a considerable gap still exists between current progress and practical clinical applications.

## 6 Conclusion

In this comprehensive review, we delve into the latest advancements in incorporating metal ions into titanium and titanium alloys through micro-arc oxidation technology, constructing biocoatings with outstanding biocompatibility. These coatings exhibit the potential to promote bone formation, enhance blood vessel formation, provide antibacterial and anti-inflammatory properties, and improve corrosion resistance. We provide detailed insights into the characteristics of commonly used metal cations and the preparation parameters of micro-arc oxidation. Additionally, we summarize the applications of various metal cations in enhancing osteogenesis, angiogenesis, antibacterial and anti-inflammatory responses, corrosion resistance, multi-ion co-doping, and the combination of different modification methods for titanium implants. We underscore the significant potential of micro-arc oxidation combined with metal cation coatings in enhancing the properties and expanding the applications of titanium and titanium alloys.

Our conclusions can be summarized as follows:

1) We present a comprehensive summary of different surface treatment technologies for modifying the surfaces of titanium and titanium alloys, analyzing the advantages and disadvantages of each method. Among various modification methods, micro-arc oxidation stands out for its ability to prepare uniform and robust nanoporous structures while stably releasing metal ions.
2) A detailed analysis of the characteristics and preparation parameters of various metal ions is provided. Different metal ions exhibit distinct biological activities; for instance, Sr demonstrates excellent osteogenic induction properties, Cu induces angiogenesis and antibacterial effects, and Zn promotes angiogenesis and osteogenesis while displaying remarkable antibacterial properties. Understanding these properties maximizes the effectiveness of their application.
3) We summarize the potential of micro-arc oxidation-added metal ion coatings in enhancing angiogenic, osteogenic, antibacterial, anti-inflammatory, and corrosion resistance activities. Optimal concentrations of metal ions for exerting the most significant biological effects are highlighted, and multi-ion co-doping is discussed as a strategy to further enhance the biological activity of titanium and titanium alloys.
4) The advantage of combining various modification methods over relying solely on micro-arc oxidation technology is discussed to improve the biological properties of titanium and titanium alloy surface coatings.
5) Despite substantial progress in the study of micro-arc oxidation coatings on titanium and titanium alloys, challenges remain. Specific concentrations of released metal ions in the coating and the mechanisms governing metal ion release require further investigation to advance the application of titanium and titanium alloys in the medical field.

In conclusion, by addressing the limitations of titanium implants in orthopedic and dental applications, optimizing their performance becomes feasible. This optimization involves tailoring the biological functions of titanium and titanium alloys based on micro-arc oxidation surfaces, encompassing osteogenesis, angiogenesis, antibacterial, anti-inflammatory, and corrosion resistance. Micro-arc oxide coatings on titanium and titanium alloys emerge as an appealing and promising strategy for enhancing bone integration in titanium implants. As micro-arc oxide coatings progress from single-element addition to multi-element doping and multi-technology combinations, coupled with an increased understanding of the mechanisms driving the biological actions of metal ion coatings, we gain a better approach to address the biological activity of titanium and titanium alloys, ultimately translating these advancements into clinical applications.
