# Supplementary material for: Advancements in incorporating metal ions onto the surface of biomedical titanium and its alloys via micro-arc oxidation: a research review
Source: Front Chem. 2024 Feb 22;12:1353950. doi: 10.3389/fchem.2024.1353950 (PMC10917964; doi:10.3389/fchem.2024.1353950)
Supplement: Supplementary file 1 [file DataSheet1.docx]

Supplementary Material

# Supplementary Figures and Tables

Table 1. Advantages and disadvantages of using different modification techniques to make coatings on the surface of titanium and its alloys.

| **Technique** | **Theory** | **Advantages** | **Disadvantages** | **Ref** |
| --- | --- | --- | --- | --- |
| Micro-arc Oxidation (MAO) | By adjusting the electrolyte and the corresponding voltage or current parameters, a rough and firmly adhering oxide ceramic layer is formed on the surface of titanium and its alloy by using the instantaneous high temperature effect generated by arc discharge | Featuring a straightforward process and exceptional treatment efficiency, this method facilitates the construction of a nanoscale porous structure. It enables the sustained and stable release of metal ions over an extended period. The coating exhibits robust bonding strength, along with notable wear and corrosion resistance | The electrolyte temperature increases rapidly and requires cooling. | (Xiu et al., 2016; Wang et al., 2021) |
| Hydro-thermal Treatment | Hydrothermal treatment entails immersing the specimen in a solution containing the desired dopant and heating the sealed high-pressure vessel to specific time and temperature parameters, reaching the melting point of the target metal. This process results in the creation of a coating on the surface of the specimen. | The production process doesn't necessitate high temperatures, is capable of creating nanoscale structures, and offers control over the composition of the coating. | Limited mechanical properties of the coatings | (Bhatti et al., 2022) |
| Physical Vapor Deposition (PVD) | A stable coating is formed on the surface by depositing atoms onto the treated object. | Improved environmental protection, Enhanced corrosion resistance, Uniform and compact coating | Insufficient adhesion | (Zhao et al., 2022) |
| Chemical Vapor Deposition (CVD) | It involves introducing two or more gaseous raw materials into a reaction chamber, where a chemical reaction takes place among these gases to generate a novel material. This newly formed material is subsequently deposited onto the surface of the substrate. | Rapid deposition rate, Thick and homogeneous coating, easily controllable composition and purity, Applicable to various substrates | Reduced sedimentation rate and increased pollution | (Mani et al., 2021) |
| Sol-Gel | The metal salt is dissolved in an organic solvent to create a homogeneous solution. Subsequently, additional components are introduced and allowed to react at a specific temperature, resulting in the formation of a gel. Finally, the product is subjected to drying processes. | Uniform coating that is easily applicable, making it suitable for coating substrates with intricate shapes | High cost, extended production time, and susceptibility to coating shrinkage | (Nichol et al., 2021) |
| Plasma Immersion Ion Implantation (PIII) | By using pulsed high voltage ionization of high-purity metal targets, the target metal ions are implanted into the titanium surface without changing the surface morphology. By changing parameters such as voltage, time, pulse frequency, pulse duration, and arc current during the injection process, the doping content of the target ions can be controlled. | This process does not impact material characterization and enables the introduction of various elements into the substrate. | Uneven injection dose, inability to construct nanostructures or microstructures, and inconsistent morphology. | (Wang et al., 2014) |
| Selective Laser Cladding (SLM) | The metal coating material to be coated is placed on the substrate surface of the coated object, and then a thin layer on the substrate surface is melted at the same time by laser irradiation, and the surface coating combined with the metal substrate is quickly solidified. | Low energy consumption, high cost-effectiveness, controllable coating composition and concentration, and reproducible coating | Rough surface and inadequate antibacterial performance | (Zhang et al., 2015) |
| Plasma Spraying | the method of using a plasma arc driven by direct current as a heat source to heat metals, alloys, and other materials to a molten or semi molten state, and spraying them onto the surface of the pre-treated material at high speed to form a firmly attached surface layer. | Suitable for materials with high hardness and a high melting point, the coating is characterized by a flat and smooth surface, resistant to oxidation, and the process is straightforward. | Elevated expenses, inadequate adhesive strength, and uneven coating | (Gu et al., 2004) |
| Magnetron Sputtering | Utilizing high-energy ions to impact the material's surface, atoms or molecules are liberated, subsequently being deposited onto the substrate's surface within a vacuum environment under magnetic field control, resulting in the formation of a thin film. | The coating exhibits high purity, robust adhesion, a dense and uniform structure, and excellent bonding properties. | The technology is intricate and expensive, necessitating subsequent heat treatment to reestablish the crystalline state of the coated structure. | (Sun et al., 2022b) |
| Sand Blast | The process involves cleaning and preparing the substrate surface through sandblasting, utilizing the force of high-speed sand flow to both cleanse and roughen the surface. | Strong coating adhesion and fast processing | Prone to generating dust and noise, susceptible to material deformation; commonly employed for treating metal surfaces. | (Velasco-Ortega et al., 2010) |
| Acid Etching | Acid corrosion is a method of using acid to corrode metals and achieve special effects | Cost-effective, straightforward, and user-friendly. | The composition, control, and treatment of acid etching liquids require a high level of professional technology. | (Aysesek et al., 2022) |
| Anodization | By applying electricity to the electrolyte, the metal material generates an oxide layer at the anode to change the surface properties of the material. | Simple production, high corrosion resistance, exceptional corrosion resistance, elevated hardness, superior wear resistance, insulation, and heat resistance. | Elevated cost, inadequate coating absorption, and limited compressive resistance. | (Michalska-Domanska et al., 2023) |

**Table 2**. Single micro-arc oxidation technology adding metal ions.

| [**Metal Ion**](javascript:;) | | **Material** | | | **Electrical Parameter** | | | | | **Electrolyte** | | Ref | |
| --- | --- | --- | --- | --- | --- | --- | --- | --- | --- | --- | --- | --- | --- |
| Ca | | | Ti-6Al-4V | | | 250 V, 660 Hz, 10% | | | | Ca(CH_3_COO)_2_, NaH_2_PO_4_·2H_2_O | | (Jo et al., 2023) | |
| Ca | | | pure-Ti plate | | | 100/500/1000Hz,20%/60% | | | | Ca(CH_3_COO)_2_, NaH_2_PO_4_ | | (Abbas et al., 2023) | |
| Ca | | | pure-Ti (IV) | | | 20%, 320 V, 50 Hz | | | | C_7_H_7_CaO_6_P·2H_2_O, Ca(CH_3_COO)·2H_2_O, EDTA-Na ,C_10_H_16_N_2_O_8_ | | (Alemayehu et al., 2023) | |
| Ca | | | Ti-25Ta-10Zr-15Nb,Ti-25Ta-20Zr-30Nb | | | 300 V, 2.5 A, 1 min | | | | C_4_H_6_CaO_4_·H_2_0, C_3_H_7_Na_2_O_6_P·5H_2_0 C_4_H_6_MgO_4_·4H_2_O | | (Kuroda et al., 2023) | |
| Ca | | | pure-Ti plate | | | 420v,50Hz,0/5/10/15min | | | | Ca(CH_3_COO)_2_·H_2_O,NaH_2_PO_4_·2H_2_O | | (Zhou et al., 2019) | |
| Ca | | | pure-Ti plate (II) | | | 190–600 V,660 Hz,10% | | | | Ca(CH_3_COO)_2_·H_2_O, CaC_3_H_7_O_6_P | | (Li et al., 2004) | |
| Ca | | | pure-Ti plate (II) | | | | | 300V,1min | | Ca(CH_3_COO)_2_·H_2_O, C_3_H_7_Na_2_O_6_P·5H_2_O | | (Ribeiro et al., 2015) | |
| Ca+Sr | | | pure-Ti plate (TA2) | | | | | 440V,600Hz, 9%, 5 min | | CaB_4_O_7_, SrB_4_O_7_, Na_2_B_4_O_7_ | | (Li et al., 2020b) | |
| Ca+Mg+Cu | | | Ti(Details unknown) | | | | | 500 V/575 V/ 600V,3 min | | Ca(NO_3_)_2_·4H_2_O, Mg(NO_3_)_2_·6H_2_O, Cu(NO_3_)_2_·3H_2_O | | (Rokosz et al., 2018) | |
| Ca+Mg+P | | | Ti-25Ta-10Zr-15Nb Ti-25Ta-20Zr-30Nb | | | | | 300V, 2.5 A, 1 min | | MgC_4_H_6_O_4_·4H_2_O, Ca(CH_3_COO)_2_·H_2_O, C_3_H_7_Na_2_O_6_P·5H_2_0 | | (Kuroda et al., 2023) | |
| Ca+Si | | | Ti-20Ta | | | | | 250/300V,50Hz,50%,5 min | | Na_2_(EDTA), Ca(CH_3_COO)_2_·H_2_O, NaOH, Na_2_SiO_3_·9H_2_O | | (Huang et al., 2018b) | |
| Ca+Sr | | | Ti-6Al-4V | | | | | 1Hz,20, 30, 40 A·dm^-2^,5/10min | | (CH3COO)_2_Ca·H_2_O, (CH3COO)_2_Sr·1/2H_2_O, C_3_H_5_(OH)_2_PO_4_Na_2_ | | (van Hengel et al., 2021a) | |
| Sr | | | pure-Ti plate | | | | | 550V,15min,15%,1000Hz | | Sr (CH_3_COO)_2_, Ca (CH_3_COO)_2_, C_3_H_7_Na_2_O_6_P·5(H_2_O) | | (Shen et al., 2022) | |
| Sr | | | pure-Ti plate | | | | | 500V, 100Hz, 26%, 10min | | (CH_3_COO)_2_Ca·H_2_O, (CH_3_COO)_2_Sr·1/2H_2_O, C_3_H_5_(OH)_2_PO_4_Na_2_ | | (Yan et al., 2013) | |
| Sr | | | pure-Ti plate | | | | | 420V,12mA, 10min | | Ca(CH_3_COO)_2_·H_2_O, C_4_H_8_O_5_Sr, C_3_H_7_CaO_6_P | | (Sato et al., 2016) | |
| Sr | | | pure-Ti plate (II) | | | | | 450V, 5min | | (CH_3_COO)_2_Ca, NaH_2_PO_4_, Sr (CH_3_COO)_2_ | | (Chung and Long, 2011) | |
| Sr+Ag | | | pure-Ti plate (TA2) | | | | | 250V,3A,800Hz,80%,10 min | | C_3_H_7_Na_2_O_6_P·5H_2_O, AgNO_3_, H_2_PtCl_6_, (CH_3_COO)_2_ Sr | | (Wang et al., 2023) | |
| Sr+Ag | | | pure-Ti plate (IV) | | | | | 300V,3A,800Hz,10 min | | (CH_3_COO)_2_ Sr, AgNO_3_ | | (Zhang et al., 2021b) | |
| Zn | | | Ti-6Al-4V | | | | | 110 mA/cm2, 2000 Hz and 35 % | | EDTA-ZnNa_2_, KOH | | (Liu et al., 2024) | |
| Zn | | | pure-Ti plate | | | | | 360V,200Hz,30%,2 min | | Ca(CH_3_COO)_2_·H2O, NaH_2_PO_4_·5H_2_0,  Zn (CH_3_COO)_2_·2H_2_O | | (Yu et al., 2023) | |
| Zn | | | pure-Ti plate (TA1) | | | | | 16.5A·dm^-2^, 800 Hz, 10%, 4 min | | C_4_H_6_O_4_Ca·H_2_O, C_3_H_7_Na_2_O_6_P·5H_2_O,  Zn (CH_3_COO)_2_·2H_2_O | | (Hu et al., 2012) | |
| Zn | | | Ti-6Al-4V | | | | | 110mA·cm^-2^, 2000Hz, 35% | | C_10_H_12_N_2_O_8_ZnNa_2_•2H_2_O, KOH, C₆HOP₆ | | (Wang et al., 2018) | |
| Zn | | | pure-Ti plate | | | | | 350-500 V, 600 Hz, 8%, 7 min | | Na_2_ (EDTA), Ca (CH_3_COO)_2_·H_2_O, NaOH, H_2_O_2_, Ca(H_2_PO_4_)_2_·H_2_O, Na_2_SiO_3_·9H_2_O, Zn (CH_3_COO)_2_ | | (Du et al., 2018) | |
| Zn | | | pure-Ti plate (II), Ti-40Nb | | | | | 200-300v,50HZ,10min | | Ca_10_(PO_4_)_6_(OH)_2_, Ca_9.9_Zn_0.1_(PO_4_)_6_(OH)_2_, Ca_9.9_Cu_0.1_(PO_4_)_6_(OH)_2_, CaCO_3_, H_3_PO_4_ | | (Sun et al., 2022a) | |
| Zn+Cu | | | pure-Ti plate | | | | | 360V, 200Hz, 30%, 2 min | | C_4_H_6_CAO_4_H_2_O, C_3_H_7_NA_2_O_6_P·5(H_2_O),  Zn (CH_3_COO)_2_·2H_2_O | | (Komarova et al., 2020a) | |
| Mg | | | Ti6Al4V | | | | | 50mA·cm^-2^, 35%,2000Hz,3 min | | C_10_H_12_N_2_O_8_Na_2_Mg, Na_2_Mg (EDTA), Na_2_MgY, KOH | | (Zhang et al., 2021a) | |
| Mg | | | Ti-6Al-4V, Ti-35Nb-7Zr-5Ta | | | | | 60min,20V,2A, 30s, 200V, 2A | | HF, Mg (CH_3_COO)_2_.4H_2_O | | (Reis et al., 2020) | |
| Mg | | | pure-Ti plate (II) | | | | | 250V, 5min | | Ca(CH_3_COO)_2_·H_2_O, Na_2_(EDTA)·2H_2_O,  Na_2_SiO_3_·9H_2_O, MgCl_2_·6H_2_O | | (Li et al., 2018b) | |
| Mg | | | Ti-6Al-4V | | | | | 420V,50Hz, 10min, | | Ca (CH_3_COO)_2_·H_2_O, NaH_2_PO_4_·2H_2_O, C_4_H_6_ MgO_4_·4H_2_O | | (Li et al., 2020a) | |
| Mg | | | pure-Ti plate (II) | | | | | 300v,30s | | Ca(CH_3_COO)_2_·H_2_O,C_3_H_7_Na_2_O_6_P, MgO | | (Fan et al., 2023) | |
| Mg+Cu+F | | | pure-Ti | | | | | 1000Hz,30%,6 min | | Ca[HOCH_2_(CHOH)_4_COO]_2_,(NaPO₃)₆, Mg(C_6_H_11_O_7_)2,Cu(C_6_H_11_O_7_)2, NaF | | (Zhao et al., 2019) | |
| Cu | | | pure-Ti plate (II) | | | | | 250V,50Hz, 50%, 5 min | | Na_2_(EDTA), NaOH,  Ca(CH3COO)_2_·H_2_O, Na_2_SiO_3_·9H_2_O, CuSO_4_·5H_2_O | | (Huang et al., 2018a) | |
| Cu | | | pure-Ti plate (II) | | | | | 270V,5min | | Na_2_(EDTA), Ca(CH_3_COO)_2_·H_2_O, Na_2_SiO_3_·9H_2_O,NaOH | | (Huang et al., 2019) | |
| Cu | | | pure-Ti plate (Cp Ti, TA85II) | | | | | 20A·dm^-2^,5min | | NaH_2_PO_3_, NaOH, Cu nanoparticle | | (Zhang et al., 2018) | |
| Cu+Si | | | pure-Ti plate | | | | | 3.25A·dm^-2^,30%,800Hz,5min | | Ca (CH_3_COO)_2_·H_2_O, NaH_2_PO_4_, Cu (CH_3_COO)_2_·H_2_O Na_2_SiO_3_·9H_2_O | | (He et al., 2020) | |
| Cu+Ag | | | pure-Ti plate (II) | | | | | 400V,251Am^−2^,10 min | | CuCl_2_, AgNO_3_ | | (Shimabukuro et al., 2020) | |
| Cu | | | Ti-30Nb-5Mo | | | | | 1 min, 2.5 A, 300 V | | (CH_3_COO)_2_Ca·H_2_O, C_3_H_7_Na_2_O_6_P·5H_2_O, (CH_3_COO)_2_Mg·4H_2_O, CuCl_2_ | | (Cardoso et al., 2023) | |
| Cu | | | pure-Ti | | | | | 80 A·cm^−2^, 600 Hz ,10% | | Ca (CH_3_COO)_2_, NaH_2_PO_4_·2H_2_O, Na_2_Cu–EDTA | | (Hu et al., 2023) | |
| Co+Sr+F | | | pure-Ti plate | | | | | 410 V, 100 Hz, 26%, 5 min | | C_4_H_6_O_4_Ca, C_4_H_6_CoO_4_, C_3_H_7_Na_2_O_6_P·5H_2_O | | (Zhou and Zhao, 2016a; b) | |
| Co+Cu | | | pure-Ti plate | | | | | 450V,1000Hz,6min | | Ca(CH₃COO)₂·H₂O, Cu(CH_3_COO)_2_·H_2_O,(CH_3_COO)_2_Co·4H_2_O | | (Zhao et al., 2021) | |
| Ag+Sr | | | pure-Ti plate | | | | | 300V,800Hz,10min | | (CH_3_COO)_2_ Sr, AgNO_3_ | | (Zhang et al., 2021b) | |
| Ag | | | pure-Ti, Ti-6Al-4V | | | | | 300v,5min | | Na_2_HPO_4_, AgC_2_H_3_O_2_ | | (Maj et al., 2023) | |
| Ag | | | pure-Ti plate (II) | | | | | 251Am^−2^,10min | | AgNO_3_ | | (Shimabukuro et al., 2019) | |
| Ag | | | pure-Ti plate | | | | | 220V,5min,4%,500Hz | | NaOH, AgNO_3_ | | (Jia et al., 2016) | |
| Ag | | | pure-Ti plate (TA1) | | | | | 280-320V,300 Hz,50%,6 min | | Ca (CH_3_COO)_2_, NaH_2_PO_4_, AgNO_3_ | | (Zhang et al., 2020a) | |
| Mn | | | Ti-6Al-4V | | | | | 280V,3min | | Ca (CH_3_COO)_2_, C_3_H_7_CaO_6_P, Mn (CH_3_COO)_2_, Na_2_SiO_3_ | | (Kang et al., 2018) | |
| Mn+Ca/P | | | pure-Ti plate | | | | | 100mA·cm^2^, 600Hz and 10 min | | Na_2_Mn (EDTA), Ca(C_2_H_3_O_2_)_2_ | | (Zhang et al., 2020b) | |
| Fe | | | pure-Ti plate | | | | | 500V,500Hz, 7.5%,3min | | (CH_3_COO)_2_Ca, C_3_H_7_Na_2_O_6_P·XH_2_O, CH₃COOH, Fe_3_O_4_–NH_2_NPs | | (Li et al., 2019) | |
| Fe | | | pure-Ti plate | | | | | 500V, 500Hz, 7.5% | | (CH_3_COO)_2_Ca, C_3_H_7_Na_2_O_6_P·XH_2_O, NaFe (EDTA) | | (Li et al., 2018a) | |
| Li | | | Pure- Ti wire(I) | | | | | 450V,3min | | LiCl, NaH_2_PO_4_, Ca(CH_3_COO)_2_ | | (Liu et al., 2018) | |
| Li | | | pure-Ti plate | | | | | 0.8A,3min, 400V | | Na_2_SiO_3_, KOH, LiCH_3_COO | | (Peng et al., 2021) | |
| Zr | | | pure-Ti plate | | | | | 50mA·cm^2^, 30℃ | | Zr(SO_4_)_2_, Ca(H_2_PO_4_)_2_ | | (Kaluderovic et al., 2014) | |
|  | | |  | | |  | | | |  | |  | |
|  | | |  | | | | |  | |  | |  | |

**Table 3.** Biological effects and mechanisms of metal cations on osteogenesis

| Metal Ion | Method | Result | Concentration of Ions | | Ref |
| --- | --- | --- | --- | --- | --- |
| Ca | HMSC | Enhanced cytocompatibility, there was a significant improvement in ALP activity, and bone formation within and around the scaffold was markedly increased. | | 0.065M Ca | (Xiu et al., 2016) |
| Ca | MG63 and HOS | the presence of Ca/p increases surface roughness and enhances bone formation. | | 0.15M Ca | (Li et al., 2004) |
| Ca | L929, Human primary osteoblast | The Ca-doped surface led to an elevation in the secretion of IFN-γ by cells, bolstered inflammation regulatory capabilities, heightened osteoblast differentiation, as well as reconfigured cytoskeletal organization and cell migration. | | 0.35 M Ca | (Ribeiro et al., 2015) |
| Ca+ Si | RAW264.7, SaOS-2 | Ti-Ta metal composite treated with Ca coating has relatively low modulus, it had enhanced osteogenic ability and good bone immunomodulatory properties. | | 0.10 M Ca | (Huang et al., 2018b) |
| Ca+ Sr | MC3T3-E1 | The coatings enriched with Ca and Sr have demonstrated the ability to enhance osteogenic differentiation in vitro and promote enhanced osseointegration strength in vivo. | | 0.15M Ca,1M Sr | (van Hengel et al., 2021a) |
| Sr | MC3T3-E1, RAW264.7 | low Sr coating facilitates the proliferation and osteogenic differentiation of MC3T3-E1 cells, while a high Sr coating enhances early bone integration through the anti-oxidative stress pathway. | | 25%,75%,100% Sr | (Shen et al., 2022) |
| Sr | Rabbit, femur | Significant cancellous bone formation was noted within the coatings containing 10% and 20% Sr, with complete bone contact established through conduction. The newly developed lamellar bone exhibited a dense and highly organized structure, accompanied by a considerable population of mature bone cells. | | The coating containing 20% Sr significantly accelerates the formation of new bone during the initial stages of the healing process. | (Yan et al., 2013) |
| Sr | MC3T3-E1 | The coating containing Sr significantly enhanced the calcification of titanium-based materials, promoted cellular osteogenic differentiation, and improved bone integration. | | 0.1M,0.15 M Sr | (Sato et al., 2016) |
| Sr | MC3T3-E1 | The coating containing Sr primarily enhances cell growth during the later stages and establishes a more robust bond between the Sr-infused coating and osteoblasts. | | When the Sr concentration exceeded 38.9%, it notably suppressed osteoclast differentiation. | (Chung and Long, 2011) |
| Sr+Ag | MC3T3-E1 | A higher Sr content resulted in improved cytocompatibility and osteoblast proliferation, while also enhancing ALP activity. | | 18.23–21.25 wt %Sr | (Wang et al., 2023) |
| Sr+Co+F | MSC | Sr influences the activities of osteoblasts, osteoclasts, and mesenchymal stem cells, thereby regulating bone remodeling and facilitating bone formation. This implies that the incorporation of both Sr and F markedly amplifies the osteogenic differentiation of MSCs. Conversely, the inclusion of Co does not exert a substantial impact. | | - | (Zhou and Zhao, 2016b) |
| Sr+Ag | MC3T3-E1 | The Sr/Ag coating demonstrates favorable cytocompatibility, facilitating the adhesion and proliferation of MC3T3 cells. Furthermore, it promotes the osteogenic differentiation of MC3T3 cells, with Sr playing a role in stimulating the proliferation and differentiation of osteoblasts, while concurrently inhibiting the differentiation and activity of osteoclasts. | | The composition with a Sr/Ag ratio of 18.23 wt% Sr and 0.58 wt% Ag exhibited the most favorable results, whereas higher silver content (1.29 wt%) resulted in toxic effects on MC3T3 cells. | (Zhang et al., 2021b) |
| Sr+Zn | BMSC | Sr triggers elevated expression of osteoblast-related genes, enhances bone nodule formation, and augments bone mineral density. | | 0.2-1 mM Sr | (Yan et al., 2022) |
| Zn | BMSC, HUVEC | Coatings containing zinc Zn exhibited an upregulation in the expression of genes such as Runx2, Osterix, ALP, OPN, and SPP1 in BMSCs. The impact of Zn^2+^ on osteogenesis was intricately linked to the MAPK/ERK signaling pathway. | | The optimal Zn^2+^ concentration was found to be 80μM, at which extracellular factors secreted by HUVEC and BMSC were mutually conducive to their functional differentiation. | (Yu et al., 2023) |
| Zn | BMSC | The Zn^2+^ released from the Zn-containing coating enhances the gene and protein expression of BMSC, particularly ALP, thereby promoting the adhesion and proliferation of BMSC. | | The highest cumulative concentration of Zn^2+^ observed in this study was 3.62 ppm. | (Hu et al., 2012) |
| Zn | BMSC, RAW264.7 | Zn forms a uniform bond with the titanium surface, ensuring stable and controlled release. It exhibits excellent biocompatibility with BMSCs, downregulates the expression of inflammation-related genes such as CD86, iNOS, and TNF-α, and significantly promotes bone formation by BMSCs. | | The presence of Zn^2+^ at concentrations ranging from 10.91μM to 27.15μM led to enhanced proliferation and ALP activity in BMSCs. However, at a higher concentration of Zn^2+^ (128.58μM), ALP activity was inhibited. | (Sun et al., 2022a) |
| Zn | HAMMSC | The coating containing zinc exhibits a favorable impact on the development of new bone, achieved through its enhancement of bone marrow stem cell differentiation, facilitation of angiogenesis, augmentation of collagen synthesis within the extracellular matrix, and promotion of calcium phosphate deposition and mineralization onto the ECM. A conceivable mechanism underlying the bone-conductive influence of zinc-containing coatings involves the stimulation of HAMMSC migration at the implant interface in in vitro conditions. | | A lower zinc content (4.40 at. %) enhanced the capacity of HAMMSCs to undergo osteoblastic differentiation in vitro. | (Komarova et al., 2020b) |
| Mg | MC3T3-E1 | The coatings containing magnesium exhibited exceptional cell viability, adhesion, proliferation, ALP activity, extracellular matrix mineralization, and collagen secretion. | | The coating with a Mg content of 2.97 at% exhibits the highest cytocompatibility, while the cytocompatibility of the coating is compromised when the Mg content reaches 6.82 at% or higher. | (Zhang et al., 2021a) |
| Mg | RAW 264.7 | The gene expressions of BMP-2, BMP-6, and VEGF were found to be up-regulated on the Mg-coated surface. This indicates that the coating containing magnesium may potentially facilitate bone formation by enhancing the gene expressions of BMP-2, BMP-6, and VEGF in macrophages. | | 3.29 mg/ml Mg | (Li et al., 2018b) |
| Mg | BMSC | Elevated levels of Mg^2+^ have been found to be detrimental to both cell adhesion and proliferation. However, when maintained at moderate concentrations, Mg^2+^ has been shown to enhance osteogenesis by influencing substrate proteins and transcription factors. | | the bone formation effect of the coating prepared by 2g/L Mg^2+^ is optimal | (Li et al., 2020a) |
| Mg | MC3T3-E1 | The Mg-Cu-F co-doped coating can enhance the adhesion, proliferation, differentiation, mineralization, and apoptosis of MC3T3 cells via the integrin β1/FAK/ERK pathway. | | 0.05-0.3mmol/L Cu | (Zhao et al., 2019) |
| Cu | SaOS-2, RAW 264.7 | Cu-containing coatings polarize macrophages into M1 phenotype and enhance macrophage-mediated osteogenesis in vitro. The mechanism underlying macrophage-mediated osteogenesis primarily involves the release of Cu2+. | | 0.2- 2 mM Cu | (Huang et al., 2018a) |
| Cu | SaOS-2, RAW 264.7 | Cu-containing coatings enhance bone integration and the expression levels of surface markers CD11 on M1-type macrophages, as well as growth factors BMP-6, OCN and Runx-2. The microenvironment resulting from the interaction between Cu-containing coatings and macrophages promotes the proliferation and differentiation of SaOS-2 cells. These findings indicate that the release of Cu^2+^ plays a crucial role in stimulating macrophage-mediated osteogenesis. | | 0.02MCu | (Huang et al., 2019) |
| Cu | MC3T3-E1 | 0.3 CuNPs promoted the proliferation and adhesion of osteoblasts, and promoted the upregulation of osteoblast-related proteins, such as ALP, OCP and OCN of osteoblasts | | The cytotoxicity observed in 3.0CuNPs is attributed to an excess of Cu ions and direct interactions between osteoblasts and CuNPs. | (Zhang et al., 2018) |
| Cu+Co | MG63 | The Cu/Co coating is implicated in modulating the functional expression of osteoblasts on the coating surface via the Wnt/ß-catenin signaling pathway, thereby fostering the adhesion, proliferation, and differentiation of MG63 cells. | | 0.05MCu  0.05MCo | (Zhao et al., 2021) |
| Ag | MC3T3-E1 | The coating containing silver can facilitate the cellular calcification process and does not adversely impact bone differentiation. | | Achieving both antimicrobial properties and osteoblast compatibility while avoiding cytotoxicity requires a surface composition of Ag (≤1.5%) and a controlled release rate of Ag+ (≤0.04 mg m^−2^ d^−1^). Notably, a concentration of 2.5 mM Ag exhibits a pronounced inhibitory effect on cell behavior. | (Shimabukuro et al., 2019) |
| Ag+Cu | MC3T3-E1 | The Ag/Cu coating stimulates ALP activity, promotes the expression of osteogenic and angiogenic markers such as OPN, OPG, Hif-1α, VEGF, and enhances stromal mineralization in mesenchymal stromal cells | | 1.5-3.0gL^−1^ Ag NPs,3.0gL^−1^ Cu NPs | (van Hengel et al., 2020) |
| Fe | hFOB1.19 | The inclusion of Fe led to a notable increase in osteoblast proliferation, upregulation of osteoblast-associated genes, enhanced collagen secretion, and extracellular matrix mineralization. This effect was particularly pronounced in the case of osteoblasts incorporated with 4.25% Fe. | | 2.27–11.07 wt% Fe | (Li et al., 2018a) |
| Li | BMDMS, C3H10T1/2 | The liberation of lithium ions from the coating surface triggers macrophage polarization and cytokine secretion, subsequently fostering the osteogenic differentiation of murine embryonic cells. This effect is primarily achieved through the activation of the MEK/ERK signal transduction pathway, resulting in the enhancement of osteogenic properties in C3H10T1/2 cells. | | 0.01-0.05M Li | (Peng et al., 2021) |
| Zr | Osteoblast | Zr has the capability to stimulate the expression of osteocalcin and bone sialoprotein, thereby exerting a favorable impact on osteoblast morphology and the formation of cell clusters. | | contains 84% (m/m) ZrO_2_ | (Kaluderovic et al., 2014) |

**Table 4.** Biological effect and mechanism of metal cations on angiogenesis.

| Metal Ion | Method | Result | Concentration of Ions | Ref |
| --- | --- | --- | --- | --- |
| Zn | HUVECS | The coating containing zinc enhanced the expression of genes such as VEGF, CD31, FGF, PDGF, and vWF, and the angiogenic potential of the high-Zn coating surpassed that of the low-Zn coating. The potential mechanism behind zinc-induced angiogenesis involves miR-29a, a molecule prominently secreted by BMSCs, which interacts with the VASH1 target, subsequently fostering angiogenesis. | Zn^2+^ at a concentration of 60μM exhibited the most favorable angiogenic response, whereas the proliferation of HUVECs was suppressed when the Zn^2+^ concentration reached 180μM. The established safe threshold for Zn^2+^ concentration remained below 180μM. | (Yu et al., 2023) |
| Cu | EA.hy926 (HUVECS) | Cu^2+^ promotes endothelial cell migration and the expression of proteins related to angiogenesis. Additionally, BMP-2, which is secreted by osteoblasts, typically binds to BMP2R receptors found on endothelial cells. This interaction enhances angiogenesis by stimulating endothelial cell proliferation and facilitating the formation of capillary tubes. | Cu 0.01mol/L  Si0.02/0.05/0.08mol/L | (He et al., 2020) |
| Cu | HAMMSC | A Cu/CaP coating has the ability to enhance the differentiation of bone marrow stem cells, promote angiogenesis, and augment collagen formation within the extracellular matrix. | **-** | (Komarova et al., 2020a) |
| Cu | endothelial cells | Coating the surface with Cu NPs enhances the secretion of angiogenesis-related proteins and gene expression, such as VEGF, HIF-1α, VEGFR-2, and endothelial nitric oxide, in endothelial cells. This stimulation fosters and facilitates the process of angiogenesis. | The proliferation and adhesion of endothelial cells showed a positive correlation with the concentration of Cu in the coating. | (Zhang et al., 2018) |
| Cu | HUVECS | Cu enhances the expression of genes associated with angiogenesis (HIF-1α, eNOS, VEGF, and KDR) and augments the secretion of VEGF protein, thereby promoting the activity of HUVECs and ultimately facilitating angiogenesis. These outcomes can be attributed to the interaction between VEGF present in the supernatant and KDR through an autocrine mechanism, leading to the upregulation of KDR and subsequently boosting angiogenesis. | Cu 0,2,4,6wt% | (Xu et al., 2018) |
| Co+Sr+F | HUVECS | The incorporation of Co significantly elevated the expression of angiogenic factors and markers. Furthermore, it effectively mimicked a hypoxic environment by stabilizing HIF-1α, thereby activating VEGF expression crucial for neovascularization and tissue regeneration. | **-** | (Zhou and Zhao, 2016b) |
| Co | MSC | Co coating enhances gene expression and protein synthesis of HIF-1a and VEGF in mesenchymal stem cells, and it can activate VEGF expression related to neovascularization and tissue regeneration by stabilizing HIF-1α. | The incorporation of Co resulted in improved angiogenesis, and the effect was positively correlated with the amount of Co incorporated (Co^2+^ concentration  0.1>0.06>0.02) | (Zhou and Zhao, 2016a) |
| Cu+Co | MG63, HOS | The gradual release of Cu and Co from the coating exhibits favorable biological activity, fostering the development of a network-like structure on the implant's surface. This structure facilitates the binding of plasma proteins within the medium, consequently fostering osteoblast proliferation. | 0.05 M Cu and 0.05 M Co were utilized. Low concentrations (0.1% w/w) of Cu were found to enhance HOS cell viability and stimulate HOS cell proliferation, whereas higher concentrations (2.5% and 1% w/w) of Cu exhibited cytotoxic effects. | (Zhao et al., 2021) |
| Li | HUVECS | Li coating enhanced transpore migration, formation of vasolike structure, and up-regulated the expression of HIF-1α, PDGF and VEGF genes. The Li-containing coating can polarize macrophages and effectively promote the angiogenesis of HUVEC in vitro. | 0.01-0.05M Li | (Peng et al., 2021) |
| Sr | HUVECS | Sr^2+^ enhances VEGF and Ang-1 secretion in HUVECS and BMSC co-culture systems, demonstrating the potential to create an angiogenic microenvironment conducive to osteogenesis at an early stage | 0.2-1 mM Sr | (Yan et al., 2022) |
|  |  |  |  |  |

**Table 5.** Antibacterial biological effect and mechanism of metal cations.

| Metal Ion | Method | Result | Concentration of Ions | | Ref |
| --- | --- | --- | --- | --- | --- |
| Zn | Staphylococcus aureus | It might be due to the fact that Zn-5.83 wt% presented more significant bactericidal effect on planktonic bacteria than Zn-2.86 wt% and Zn-8.81 wt% via releasing more Zn ions to the solution. Thus, the higher concentration of released Zn ions, the better antibacterial effect of samples. | | Zn-containing coatings especially Zn-5.83 wt% induces the strongest oxidative stress on S. aureus | (Liu et al., 2024) |
| Zn | Staphylococcus aureus, Escherichia coli | The Zn-doped coating exhibited a greater inhibitory effect against Staphylococcus aureus compared to Escherichia coli cells, and this inhibitory effect was even more pronounced in coatings with higher Zn content. The primary antibacterial mechanism attributed to ZnO is the generation of ROS. | | The antibacterial effect of the Zn 9.3±0.8wt.% coating is enhanced | (Hu et al., 2012) |
| Zn/Ca | Staphylococcus aureus, Escherichia coli | The antibacterial efficacy of the Ca/P coating against Escherichia coli surpasses that of the Zn coating. However, the antibacterial potency of the Ca/P coating against Staphylococcus aureus falls short of that exhibited by the Zn coating. The presence of Ca and P elements enhances the antibacterial effectiveness against Escherichia coli, with Staphylococcus aureus displaying heightened sensitivity to Zn in comparison to Escherichia coli. | | The concentration of Zn^2+^ is altered through voltage adjustments, with the most effective antibacterial impact of Zn^2+^ observed at a voltage of 350V. | (Du et al., 2018) |
| Zn/Cu | Staphylococcus aureus | Coatings containing Zn and Cu exhibit antibacterial effects against Staphylococcus aureus, with the antibacterial efficacy ranked as follows: Zn > Cu. | | - | (Komarova et al., 2020b) |
| Cu+Mg+F | Staphylococcus aureus | Antibacterial activity ranking: Cu>Mg+Cu+F>F>Mg | | A Cu^2+^ concentration exceeding 0.5 mmol/L exhibits antibacterial effects. | (Zhao et al., 2019) |
| Cu | Staphylococcus aureus | Surfaces containing copper enhance the bactericidal capabilities of macrophages, while Cu^2+^ controls the macrophage phenotype, resulting in the elimination of bacteria through the regulation of ROS and NO production. | | The ultimate concentration of Cu^2+^, which is 0.4113 ppm, augments the macrophages' capability to engulf and eliminate bacteria. | (Huang et al., 2018a) |
| Cu | Staphylococcus aureus | Coatings containing copper enhance the bactericidal efficacy against Staphylococcus aureus. The optimal concentration of Cu^2+^ needed for the bactericidal effect mediated by macrophages is lower than that required for the direct eradication of bacteria. The mechanism underlying the augmented bactericidal activity of macrophages in the presence of copper may be attributed to the direct toxic impact of Cu^2+^ on bacteria through Fenton chemistry, which involves the generation of ROS triggered by Cu^2+^ interaction with mitochondrial vesicles. | | 0.02M Cu | (Huang et al., 2019) |
| Cu | Staphylococcus aureus | The coating of Cu NPs exhibited remarkable antibacterial activity attributed to the synergistic effects of both controlled release-mediated bactericidal action and direct contact-based bactericidal action. | | Coatings containing 0.3 and 3.0Cu NPs exhibit enhanced antimicrobial properties. | (Zhang et al., 2018) |
| Cu+Ag | Escherichia coli | The coatings doped with Ag and Cu demonstrate instances of transient effects that contribute to their antibacterial activity against E. coli. | | 2.5 mM Ag,2.5 mM Cu | (Shimabukuro et al., 2020) |
| Cu | Staphylococcus aureus, Enterococcus faecalis, Pseudomonas aeruginosa bacteria strains, and Candida albicans fungus | Cu incorporation increased the antimicrobial activity of the coatings, inhibiting the growth of Staphylococcus aureus, Enterococcus faecalis, Pseudomonas aeruginosa bacteria strains, and Candida albicans fungus by approximately 44%, 37%, 19%, and 41%, respectively | | Cu (up to 3.5 mM) did not affect the differentiation of AMSCs into the osteogenic lineage | (Cardoso et al., 2023) |
| Ag+Cu | MRSA | The synergy between Ag and Cu resulted in an augmented bactericidal activity against MRSA. | | 75%Ag NP, 25%Cu NP | (van Hengel et al., 2020) |
| Zn/Ag/Cu | Staphylococcus aureus, Escherichia coli | The incorporation of Ag, Cu, and Zn can enhance antibacterial efficacy, potentially through a mechanism involving the diffusion of released ions from the implant onto the bacterial cell wall and their subsequent penetration into the bacteria. Concurrently, the presence of highly reactive ROS contributes to the disruption and lysis of the bacterial cell wall. | | 100 Ag,93% Cu,73% Zn | (van Hengel et al., 2021b) |
| Zn/Ca/P/F | Porphyromonas gingivalis | Replacing CaP coatings with Zn/F coatings can lead to an enhanced bacterial-killing rate, potentially achieved by hindering ATP synthesis upon absorption of Zn by bacterial cells. Additionally, fluoride has the capability to disrupt bacterial enzymes and membrane functions. | | Ca:P:Zn:F=20:12:5:5 | (Kulkarni Aranya et al., 2017) |
| Cu | Staphylococcus aureus | Both Cu and CuSi coatings demonstrate the capability to efficiently eliminate Staphylococcus aureus, yielding remarkable antibacterial outcomes. The liberated Cu^2+^ ions have the ability to attach to the bacterial membrane's surface, leading to the disruption of respiratory enzymes or the generation of reactive oxygen species. This results in cellular lysis, leakage of cytoplasmic content, and eventual bacterial demise. | | Cu 0.01mol/L, Si 0.02/0.05/0.08mol/L | (He et al., 2020) |
| Sr+Co+F | Staphylococcus aureus, Escherichia coli | The inclusion of Sr, Co, and F can confer antibacterial capability upon the implant, effective against both Gram-positive and Gram-negative bacteria. Notably, the incorporation of F exhibits greater antibacterial efficacy compared to Co, influencing bacterial metabolism through direct interaction with enzyme inhibitors and the inhibition of proton-translocating F-ATP ase. | | - | (Zhou and Zhao, 2016b) |
| Cu+Co | Staphylococcus aureus | The Cu/Co coating demonstrated inhibition in the proliferation of Staphylococcus aureus, with the antibacterial efficacy ranking as Cu/Co > Cu > Co. This indicated that the antibacterial performance of the Cu-containing coating surpassed that of the other coatings. This enhancement could be attributed to the bactericidal impact achieved through the "contact sterilization" effect, resulting from the release of copper ions from the coating. | | 0.05MCu  0.05MCo | (Zhao et al., 2021) |
| Ag+Sr | Staphylococcus aureus | The coatings containing 18.5 wt% Sr and 0.58 wt% Ag exhibited robust antibacterial activity over both short and extended periods. Furthermore, the direct contact with Ag significantly contributed to the efficacy of the bactericidal mechanism. | | When the Ag^+^ concentration reached 0.58 wt%, the coating exhibited favorable short-term antibacterial activity (100.0%) as well as sustained long-term antibacterial activity (77.6%). | (Zhang et al., 2021b) |
| Ag | Staphylococcus aureus, Escherichia coli | The Ag-containing coating showed good antibacterial effect on both Staphylococcus aureus and Escherichia coli, and the antibacterial effect was proportional to the concentration | | The coating containing Ag (≥0.004 mM Ag) showed good antibacterial effect on Escherichia coli, and the samples with Ag^+^ concentration above 0.05 mM also showed good antibacterial effect on Staphylococcus aureus | (Shimabukuro et al., 2019) |
| Ag | Staphylococcus aureus | By effectively combining the physical trapping effect of micropores with the chemical bactericidal properties of Ag particles and ions, an efficient "trap-killing" antibacterial mechanism was achieved. | | Significantly elevated Ag^+^ concentrations (exceeding 8.57 mg/cm2) for continuous and enduring release. | (Jia et al., 2016) |
| Ag+Ca | Escherichia coli | The growth of E. coli was effectively inhibited on both Ag+Ca/P coatings, with an antibacterial rate exceeding 99.9%. The bactericidal activity of Ag was attributed to its interaction with the bacterial membrane. This interaction led to destabilization of the membrane potential and depletion of intracellular ATP levels, ultimately resulting in bacterial cell death. | | Ag^+^ concentration that cells can tolerate ranges from 1.6 to 31.75 μg, while the released Ag^+^ concentration in this study falls within the range of 0.00025 to 0.0003 mol/L. | (Zhang et al., 2020a) |

Table 6. Corrosion resistance effect of metal cations.

| **Metal Ion** | **Method** | **Result** | **Ref** |
| --- | --- | --- | --- |
| Cu | MAO | The formation of TiO_2_ coating improves the corrosion resistance of the Ti substrate, while copper incorporation is insensitive to the effect of corrosion resistance | (Hu et al., 2023) |
| Ca | PEO | PEO-coatings possess the high corrosion resistance in comparison with uncoated titanium, reducing the corrosion current density by more than 15 times. | (Mashtalyar et al., 2020) |
| Zn | MAO + ultrasonic vibration (UV) | Due to the sealing effect of Zn-doped Ca/P-rich compounds, resulting in the beneficial role of a reduced porosity for the anti-corrosion property of the ceramic coating | (Lv et al., 2021a) |
| Zr | PEO | The sample coated with 3g/l ZrO_2_ nanoparticles showed the peak corrosion resistance compared to its counterparts. | (Nikoomanzari et al., 2020) |
| Zn/Zr | PEO | The corrosion resistance of the coating increased significantly with using of ZrO_2_, ZnO nanoparticles due to the improvements in coating. The lowest corrosion rate was obtained for the sample containing ZnO NP (1.89×10^−8^Acm^−2^) and the mixed ZnO and ZrO_2_ nanoparticles (2.37×10^−8^Acm^−2^), which was almost 14 times lower than that of the substrate). | (Nadimi and Dehghanian, 2021) |
| Na | PEO | The results revealed that use of a sodium phosphate additive led to formation of a coating (4.33 µm thick) with the most suitable microstructure and highest corrosion resistance (4.36×10^6^Ω cm^2^), which was 53 times higher than that of uncoated Ti. | (Molaei et al., 2019) |
| Ag+Zn | MAO | The synergistic effect of Ag and ZnO enhances the corrosion resistance and biological performances of titania. | (Lv et al., 2021b) |

## Supplementary Figures

**Supplementary Figure 1.** Graphical Abstract


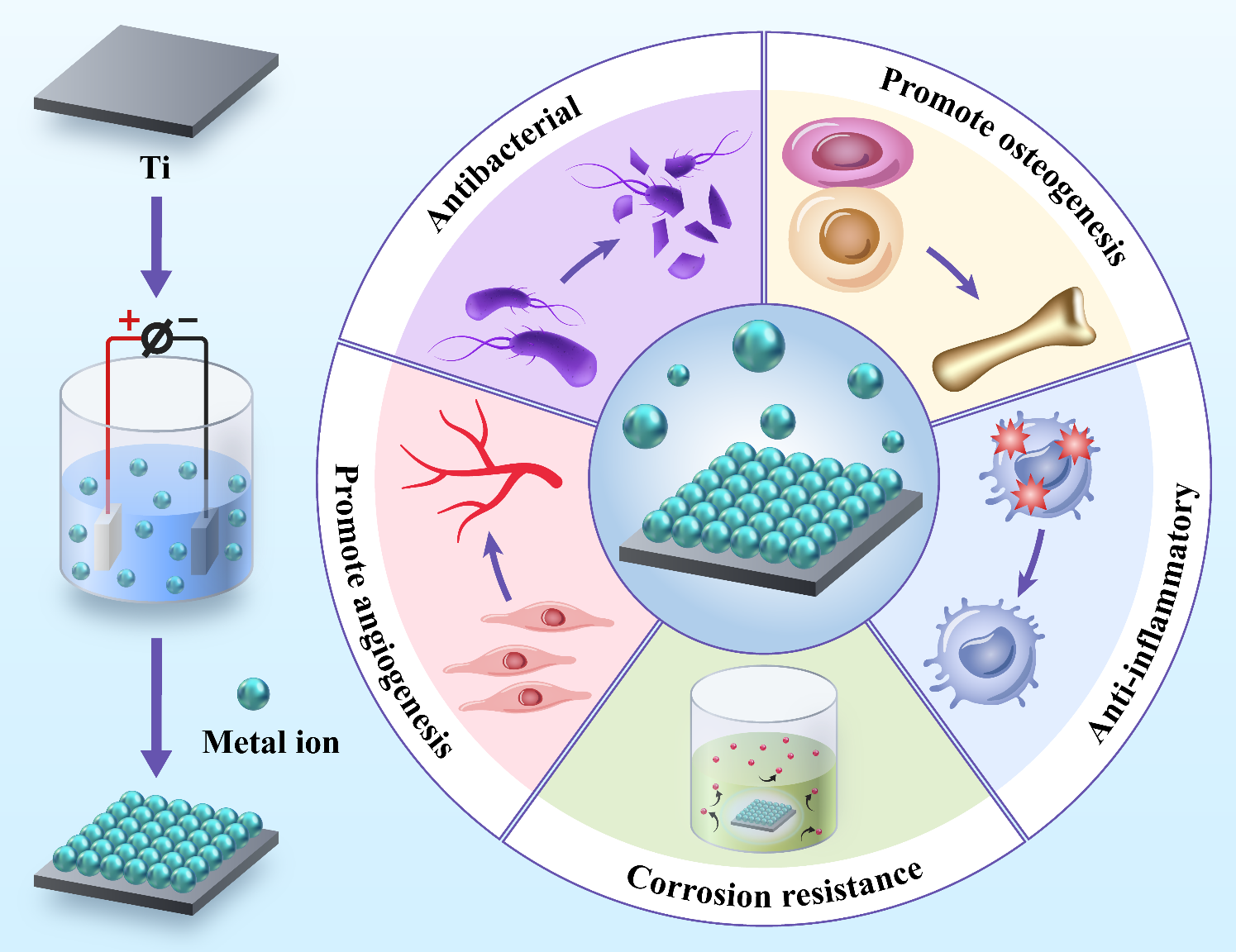


Abbas, A., Kung, H.P., and Lin, H.C. (2023). Effects of Electrical Parameters on Micro-Arc Oxidation Coatings on Pure Titanium. *Micromachines (Basel)* 14(10). doi: 10.3390/mi14101950.

Alemayehu, D.B., Todoh, M., Hsieh, J.H., Li, C., and Huang, S.J. (2023). Improving Pure Titanium's Biological and Mechanical Characteristics through ECAP and Micro-Arc Oxidation Processes. *Micromachines (Basel)* 14(8). doi: 10.3390/mi14081541.

Aysesek, N., Arisan, V., Balcioglu, N.B., Erol, A., Kuruoglu, F., Tekkesin, M.S., et al. (2022). Boron- and Boric Acid-Treated Titanium Implant Surfaces in Sheep Tibia: A Histologic, Histomorphometric and Mechanical Study. *Bioengineering (Basel)* 9(11). doi: 10.3390/bioengineering9110705.

Bhatti, M.A., Gilani, S.J., Shah, A.A., Channa, I.A., Almani, K.F., Chandio, A.D., et al. (2022). Effective Removal of Methylene Blue by Surface Alteration of TiO(2) with Ficus Carica Leaf Extract under Visible Light. *Nanomaterials (Basel)* 12(16). doi: 10.3390/nano12162766.

Cardoso, G.C., Barbaro, K., Kuroda, P.A.B., De Bonis, A., Teghil, R., Krasnyuk, I.I., Jr., et al. (2023). Antimicrobial Cu-Doped TiO(2) Coatings on the beta Ti-30Nb-5Mo Alloy by Micro-Arc Oxidation. *Materials (Basel)* 17(1). doi: 10.3390/ma17010156.

Chung, C.J., and Long, H.Y. (2011). Systematic strontium substitution in hydroxyapatite coatings on titanium via micro-arc treatment and their osteoblast/osteoclast responses. *Acta Biomater* 7(11)**,** 4081-4087. doi: 10.1016/j.actbio.2011.07.004.

Du, Q., Wei, D., Wang, Y., Cheng, S., Liu, S., Zhou, Y., et al. (2018). The effect of applied voltages on the structure, apatite-inducing ability and antibacterial ability of micro arc oxidation coating formed on titanium surface. *Bioact Mater* 3(4)**,** 426-433. doi: 10.1016/j.bioactmat.2018.06.001.

Fan, X., Du, J., Li, Y., Duan, K., and Liu, G. (2023). Electrophoretic deposition of magnesium oxide coating on micro-arc oxidized titanium for antibacterial activity and biocompatibility. *J Orthop Surg Res* 18(1)**,** 901. doi: 10.1186/s13018-023-04390-4.

Gu, Y.W., Khor, K.A., Pan, D., and Cheang, P. (2004). Activity of plasma sprayed yttria stabilized zirconia reinforced hydroxyapatite/Ti-6Al-4V composite coatings in simulated body fluid. *Biomaterials* 25(16)**,** 3177-3185. doi: 10.1016/j.biomaterials.2003.09.101.

He, X., Zhang, G., Zhang, H., Hang, R., Huang, X., Yao, X., et al. (2020). Cu and Si co-doped microporous TiO2 coating for osseointegration by the coordinated stimulus action. *Applied Surface Science* 503. doi: 10.1016/j.apsusc.2019.144072.

Hu, H., Zhang, W., Qiao, Y., Jiang, X., Liu, X., and Ding, C. (2012). Antibacterial activity and increased bone marrow stem cell functions of Zn-incorporated TiO2 coatings on titanium. *Acta Biomater* 8(2)**,** 904-915. doi: 10.1016/j.actbio.2011.09.031.

Hu, P., Zhu, L., Tian, C., Xu, G., Zhang, X., and Cai, G. (2023). Study of Anticorrosion and Antifouling Properties of a Cu-Doped TiO(2) Coating Fabricated via Micro-Arc Oxidation. *Materials (Basel)* 17(1). doi: 10.3390/ma17010217.

Huang, Q., Li, X., Elkhooly, T.A., Liu, X., Zhang, R., Wu, H., et al. (2018a). The Cu-containing TiO2 coatings with modulatory effects on macrophage polarization and bactericidal capacity prepared by micro-arc oxidation on titanium substrates. *Colloids Surf B Biointerfaces* 170**,** 242-250. doi: 10.1016/j.colsurfb.2018.06.020.

Huang, Q., Li, X., Elkhooly, T.A., Xu, S., Liu, X., Feng, Q., et al. (2018b). The osteogenic, inflammatory and osteo-immunomodulatory performances of biomedical Ti-Ta metal-metal composite with Ca- and Si-containing bioceramic coatings. *Colloids Surf B Biointerfaces* 169**,** 49-59. doi: 10.1016/j.colsurfb.2018.05.010.

Huang, Q., Ouyang, Z., Tan, Y., Wu, H., and Liu, Y. (2019). Activating macrophages for enhanced osteogenic and bactericidal performance by Cu ion release from micro/nano-topographical coating on a titanium substrate. *Acta Biomater* 100**,** 415-426. doi: 10.1016/j.actbio.2019.09.030.

Jia, Z., Xiu, P., Li, M., Xu, X., Shi, Y., Cheng, Y., et al. (2016). Bioinspired anchoring AgNPs onto micro-nanoporous TiO2 orthopedic coatings: Trap-killing of bacteria, surface-regulated osteoblast functions and host responses. *Biomaterials* 75**,** 203-222. doi: 10.1016/j.biomaterials.2015.10.035.

Jo, W.L., Lim, Y.W., Kwon, S.Y., Bahk, J.H., Kim, J., Shin, T., et al. (2023). Non-thermal atmospheric pressure plasma treatment increases hydrophilicity and promotes cell growth on titanium alloys in vitro. *Sci Rep* 13(1)**,** 14792. doi: 10.1038/s41598-023-41905-9.

Kaluderovic, M.R., Schreckenbach, J.P., and Graf, H.L. (2014). Zirconia coated titanium for implants and their interactions with osteoblast cells. *Mater Sci Eng C Mater Biol Appl* 44**,** 254-261. doi: 10.1016/j.msec.2014.08.032.

Kang, J.I., Son, M.K., and Choe, H.C. (2018). Hydroxyapatite Coatings Containing Mn and Si on the Oxidized Ti-6Al-4V Alloy for Dental Applications. *J Nanosci Nanotechnol* 18(2)**,** 833-836. doi: 10.1166/jnn.2018.14883.

Komarova, E.G., Sharkeev, Y.P., Sedelnikova, M.B., Prosolov, K.A., Khlusov, I.A., Prymak, O., et al. (2020a). Zn- or Cu-Containing CaP-Based Coatings Formed by Micro-arc Oxidation on Titanium and Ti-40Nb Alloy: Part I-Microstructure, Composition and Properties. *Materials (Basel)* 13(18). doi: 10.3390/ma13184116.

Komarova, E.G., Sharkeev, Y.P., Sedelnikova, M.B., Prymak, O., Epple, M., Litvinova, L.S., et al. (2020b). Zn- or Cu-containing CaP-Based Coatings Formed by Micro-Arc Oxidation on Titanium and Ti-40Nb Alloy: Part II-Wettability and Biological Performance. *Materials (Basel)* 13(19). doi: 10.3390/ma13194366.

Kulkarni Aranya, A., Pushalkar, S., Zhao, M., LeGeros, R.Z., Zhang, Y., and Saxena, D. (2017). Antibacterial and bioactive coatings on titanium implant surfaces. *J Biomed Mater Res A* 105(8)**,** 2218-2227. doi: 10.1002/jbm.a.36081.

Kuroda, P.A.B., Grandini, C.R., and Afonso, C.R.M. (2023). Surface Characterization of New beta Ti-25Ta-Zr-Nb Alloys Modified by Micro-Arc Oxidation. *Materials (Basel)* 16(6). doi: 10.3390/ma16062352.

Li, K., Liu, S., Xue, Y., Zhang, L., and Han, Y. (2019). A superparamagnetic Fe(3)O(4)-TiO(2) composite coating on titanium by micro-arc oxidation for percutaneous implants. *J Mater Chem B* 7(34)**,** 5265-5276. doi: 10.1039/c9tb01096c.

Li, K., Yan, T., Xue, Y., Guo, L., Zhang, L., and Han, Y. (2018a). Intrinsically ferromagnetic Fe-doped TiO(2) coatings on titanium for accelerating osteoblast response in vitro. *J Mater Chem B* 6(36)**,** 5756-5767. doi: 10.1039/c8tb01414k.

Li, L.H., Kong, Y.M., Kim, H.W., Kim, Y.W., Kim, H.E., Heo, S.J., et al. (2004). Improved biological performance of Ti implants due to surface modification by micro-arc oxidation. *Biomaterials* 25(14)**,** 2867-2875. doi: 10.1016/j.biomaterials.2003.09.048.

Li, X., Huang, Q., Liu, L., Zhu, W., Elkhooly, T.A., Liu, Y., et al. (2018b). Reduced inflammatory response by incorporating magnesium into porous TiO2 coating on titanium substrate. *Colloids Surf B Biointerfaces* 171**,** 276-284. doi: 10.1016/j.colsurfb.2018.07.032.

Li, X., Wang, M., Zhang, W., Bai, Y., Liu, Y., Meng, J., et al. (2020a). A Magnesium-Incorporated Nanoporous Titanium Coating for Rapid Osseointegration. *Int J Nanomedicine* 15**,** 6593-6603. doi: 10.2147/IJN.S255486.

Li, Y., Wang, W., Yu, F., Wang, D., Guan, S., Li, Y., et al. (2020b). Characterization and cytocompatibility of hierarchical porous TiO(2) coatings incorporated with calcium and strontium by one-step micro-arc oxidation. *Mater Sci Eng C Mater Biol Appl* 109**,** 110610. doi: 10.1016/j.msec.2019.110610.

Liu, W., Chen, D., Jiang, G., Li, Q., Wang, Q., Cheng, M., et al. (2018). A lithium-containing nanoporous coating on entangled titanium scaffold can enhance osseointegration through Wnt/beta-catenin pathway. *Nanomedicine* 14(1)**,** 153-164. doi: 10.1016/j.nano.2017.09.006.

Liu, Y., Chen, C., Liang, T., Wang, Y., Zhao, R., Li, G., et al. (2024). In vitro long-term antibacterial performance and mechanism of Zn-doped micro-arc oxidation coatings. *Colloids Surf B Biointerfaces* 233**,** 113634. doi: 10.1016/j.colsurfb.2023.113634.

Lv, Y., Sun, S., Zhang, X., Lu, X., and Dong, Z. (2021a). Construction of multi-layered Zn-modified TiO(2) coating by ultrasound-auxiliary micro-arc oxidation: Microstructure and biological property. *Mater Sci Eng C Mater Biol Appl* 131**,** 112487. doi: 10.1016/j.msec.2021.112487.

Lv, Y., Zhang, T., Zhang, X., Fu, S., Yang, L., Dong, Z., et al. (2021b). The synergistic effect of Ag and ZnO on the microstructure, corrosion resistance and in vitro biological performance of titania coating. *Surface and Coatings Technology* 426**,** 127798. doi: <https://doi.org/10.1016/j.surfcoat.2021.127798>.

Maj, L., Fogarassy, Z., Wojtas, D., Jarzebska, A., Muhaffel, F., Sulyok, A., et al. (2023). In-situ formation of Ag nanoparticles in the MAO coating during the processing of cp-Ti. *Sci Rep* 13(1)**,** 3230. doi: 10.1038/s41598-023-29999-7.

Mani, N., Ahnood, A., Peng, D., Tong, W., Booth, M., Jones, A., et al. (2021). Single-Step Fabrication Method toward 3D Printing Composite Diamond-Titanium Interfaces for Neural Applications. *ACS Appl Mater Interfaces* 13(27)**,** 31474-31484. doi: 10.1021/acsami.1c07318.

Mashtalyar, D.V., Nadaraia, K.V., Gnedenkov, A.S., Imshinetskiy, I.M., Piatkova, M.A., Pleshkova, A.I., et al. (2020). Bioactive Coatings Formed on Titanium by Plasma Electrolytic Oxidation: Composition and Properties. *Materials (Basel)* 13(18). doi: 10.3390/ma13184121.

Michalska-Domanska, M., Prabucka, K., and Czerwinski, M. (2023). Modification of Anodic Titanium Oxide Bandgap Energy by Incorporation of Tungsten, Molybdenum, and Manganese In Situ during Anodization. *Materials (Basel)* 16(7). doi: 10.3390/ma16072707.

Molaei, M., Fattah-Alhosseini, A., and Keshavarz, M.K. (2019). Influence of different sodium-based additives on corrosion resistance of PEO coatings on pure Ti. *Journal of Asian Ceramic Societies* 7(2)**,** 247-255. doi: 10.1080/21870764.2019.1604609.

Nadimi, M., and Dehghanian, C. (2021). Incorporation of ZnO–ZrO2 nanoparticles into TiO2 coatings obtained by PEO on Ti–6Al–4V substrate and evaluation of its corrosion behavior, microstructural and antibacterial effects exposed to SBF solution. *Ceramics International* 47(23)**,** 33413-33425. doi: <https://doi.org/10.1016/j.ceramint.2021.08.248>.

Nichol, T., Callaghan, J., Townsend, R., Stockley, I., Hatton, P.V., Le Maitre, C., et al. (2021). The antimicrobial activity and biocompatibility of a controlled gentamicin-releasing single-layer sol-gel coating on hydroxyapatite-coated titanium. *Bone Joint J* 103-B(3)**,** 522-529. doi: 10.1302/0301-620X.103B3.BJJ-2020-0347.R1.

Nikoomanzari, E., Fattah-alhosseini, A., Pajohi Alamoti, M.R., and Keshavarz, M.K. (2020). Effect of ZrO2 nanoparticles addition to PEO coatings on Ti–6Al–4V substrate: Microstructural analysis, corrosion behavior and antibacterial effect of coatings in Hank's physiological solution. *Ceramics International* 46(9)**,** 13114-13124. doi: <https://doi.org/10.1016/j.ceramint.2020.02.084>.

Peng, F., Qiu, L., Yao, M., Liu, L., Zheng, Y., Wu, S., et al. (2021). A lithium-doped surface inspires immunomodulatory functions for enhanced osteointegration through PI3K/AKT signaling axis regulation. *Biomater Sci* 9(24)**,** 8202-8220. doi: 10.1039/d1bm01075a.

Reis, B.A., Fais, L.M., Ribeiro, A.L.R., and Vaz, L.G. (2020). Comparison of Ti-35Nb-7Zr-5Ta and Ti-6Al-4V hydrofluoric acid/magnesium-doped surfaces obtained by anodizing. *Heliyon* 6(8)**,** e04762. doi: 10.1016/j.heliyon.2020.e04762.

Ribeiro, A.R., Oliveira, F., Boldrini, L.C., Leite, P.E., Falagan-Lotsch, P., Linhares, A.B., et al. (2015). Micro-arc oxidation as a tool to develop multifunctional calcium-rich surfaces for dental implant applications. *Mater Sci Eng C Mater Biol Appl* 54**,** 196-206. doi: 10.1016/j.msec.2015.05.012.

Rokosz, K., Hryniewicz, T., Gaiaschi, S., Chapon, P., Raaen, S., Matysek, D., et al. (2018). Novel Porous Phosphorus(-)Calcium(-)Magnesium Coatings on Titanium with Copper or Zinc Obtained by DC Plasma Electrolytic Oxidation: Fabrication and Characterization. *Materials (Basel)* 11(9). doi: 10.3390/ma11091680.

Sato, M., Chen, P., Tsutsumi, Y., Shiota, M., Hanawa, T., and Kasugai, S. (2016). Effect of strontium ions on calcification of preosteoblasts cultured on porous calcium- and phosphate-containing titanium oxide layers formed by micro-arc oxidation. *Dent Mater J* 35(4)**,** 627-634. doi: 10.4012/dmj.2016-032.

Shen, X., Fang, K., Ru Yie, K.H., Zhou, Z., Shen, Y., Wu, S., et al. (2022). High proportion strontium-doped micro-arc oxidation coatings enhance early osseointegration of titanium in osteoporosis by anti-oxidative stress pathway. *Bioact Mater* 10**,** 405-419. doi: 10.1016/j.bioactmat.2021.08.031.

Shimabukuro, M., Hiji, A., Manaka, T., Nozaki, K., Chen, P., Ashida, M., et al. (2020). Time-Transient Effects of Silver and Copper in the Porous Titanium Dioxide Layer on Antibacterial Properties. *J Funct Biomater* 11(2). doi: 10.3390/jfb11020044.

Shimabukuro, M., Tsutsumi, Y., Yamada, R., Ashida, M., Chen, P., Doi, H., et al. (2019). Investigation of Realizing Both Antibacterial Property and Osteogenic Cell Compatibility on Titanium Surface by Simple Electrochemical Treatment. *ACS Biomater Sci Eng* 5(11)**,** 5623-5630. doi: 10.1021/acsbiomaterials.8b01058.

Sun, H., Yang, Y., Yu, L., Liu, K., Fei, Y., Guo, C., et al. (2022a). Inhibition of Inflammatory Response and Promotion of Osteogenic Activity of Zinc-Doped Micro-Arc Titanium Oxide Coatings. *ACS Omega* 7(17)**,** 14920-14932. doi: 10.1021/acsomega.2c00579.

Sun, Z., Khlusov, I.A., Evdokimov, K.E., Konishchev, M.E., Kuzmin, O.S., Khaziakhmatova, O.G., et al. (2022b). Nitrogen-doped titanium dioxide films fabricated via magnetron sputtering for vascular stent biocompatibility improvement. *J Colloid Interface Sci* 626**,** 101-112. doi: 10.1016/j.jcis.2022.06.114.

van Hengel, I.A.J., Lacin, M., Minneboo, M., Fratila-Apachitei, L.E., Apachitei, I., and Zadpoor, A.A. (2021a). The effects of plasma electrolytically oxidized layers containing Sr and Ca on the osteogenic behavior of selective laser melted Ti6Al4V porous implants. *Mater Sci Eng C Mater Biol Appl* 124**,** 112074. doi: 10.1016/j.msec.2021.112074.

van Hengel, I.A.J., Tierolf, M., Fratila-Apachitei, L.E., Apachitei, I., and Zadpoor, A.A. (2021b). Antibacterial Titanium Implants Biofunctionalized by Plasma Electrolytic Oxidation with Silver, Zinc, and Copper: A Systematic Review. *Int J Mol Sci* 22(7). doi: 10.3390/ijms22073800.

van Hengel, I.A.J., Tierolf, M., Valerio, V.P.M., Minneboo, M., Fluit, A.C., Fratila-Apachitei, L.E., et al. (2020). Self-defending additively manufactured bone implants bearing silver and copper nanoparticles. *J Mater Chem B* 8(8)**,** 1589-1602. doi: 10.1039/c9tb02434d.

Velasco-Ortega, E., Jos, A., Camean, A.M., Pato-Mourelo, J., and Segura-Egea, J.J. (2010). In vitro evaluation of cytotoxicity and genotoxicity of a commercial titanium alloy for dental implantology. *Mutat Res* 702(1)**,** 17-23. doi: 10.1016/j.mrgentox.2010.06.013.

Wang, G., Li, J., Zhang, W., Xu, L., Pan, H., Wen, J., et al. (2014). Magnesium ion implantation on a micro/nanostructured titanium surface promotes its bioactivity and osteogenic differentiation function. *Int J Nanomedicine* 9**,** 2387-2398. doi: 10.2147/IJN.S58357.

Wang, X., Mei, L., Jiang, X., Jin, M., Xu, Y., Li, J., et al. (2021). Hydroxyapatite-Coated Titanium by Micro-Arc Oxidation and Steam-Hydrothermal Treatment Promotes Osseointegration. *Front Bioeng Biotechnol* 9**,** 625877. doi: 10.3389/fbioe.2021.625877.

Wang, Y., Zeng, L., Zhang, H., Xiang, J., Zhang, S., Chang, W., et al. (2018). Investigation of Zinc and Phosphorus Elements Incorporated into Micro-Arc Oxidation Coatings Developed on Ti-6Al-4V Alloys. *Materials (Basel)* 11(3). doi: 10.3390/ma11030344.

Wang, Y.R., Yang, N.Y., Sun, H., Dong, W., Deng, J.P., Zheng, T.X., et al. (2023). The effect of strontium content on physicochemical and osteogenic property of Sr/Ag-containing TiO(2) microporous coatings. *J Biomed Mater Res B Appl Biomater* 111(4)**,** 846-857. doi: 10.1002/jbm.b.35195.

Xiu, P., Jia, Z., Lv, J., Yin, C., Cheng, Y., Zhang, K., et al. (2016). Tailored Surface Treatment of 3D Printed Porous Ti6Al4V by Microarc Oxidation for Enhanced Osseointegration via Optimized Bone In-Growth Patterns and Interlocked Bone/Implant Interface. *ACS Appl Mater Interfaces* 8(28)**,** 17964-17975. doi: 10.1021/acsami.6b05893.

Xu, X., Lu, Y., Li, S., Guo, S., He, M., Luo, K., et al. (2018). Copper-modified Ti6Al4V alloy fabricated by selective laser melting with pro-angiogenic and anti-inflammatory properties for potential guided bone regeneration applications. *Mater Sci Eng C Mater Biol Appl* 90**,** 198-210. doi: 10.1016/j.msec.2018.04.046.

Yan, J., Sun, J.F., Chu, P.K., Han, Y., and Zhang, Y.M. (2013). Bone integration capability of a series of strontium-containing hydroxyapatite coatings formed by micro-arc oxidation. *J Biomed Mater Res A* 101(9)**,** 2465-2480. doi: 10.1002/jbm.a.34548.

Yan, R., Li, J., Wu, Q., Zhang, X., Hu, L., Deng, Y., et al. (2022). Trace Element-Augmented Titanium Implant With Targeted Angiogenesis and Enhanced Osseointegration in Osteoporotic Rats. *Front Chem* 10**,** 839062. doi: 10.3389/fchem.2022.839062.

Yu, L., Yin, Y., Guo, Z., Fei, Y., Wen, X., Wang, J., et al. (2023). A functional study of zinc-titanium coatings and exploration of the intrinsic correlation between angiogenesis and osteogenesis. *J Mater Chem B* 11(14)**,** 3236-3251. doi: 10.1039/d3tb00119a.

Zhang, L., Li, B., Zhang, X., Wang, D., Zhou, L., Li, H., et al. (2020a). Biological and antibacterial properties of TiO(2) coatings containing Ca/P/Ag by one-step and two-step methods. *Biomed Microdevices* 22(2)**,** 24. doi: 10.1007/s10544-020-00482-8.

Zhang, R., Zhong, S., Zeng, L., Li, H., Zhao, R., Zhang, S., et al. (2021a). Novel Mg-Incorporated Micro-Arc Oxidation Coatings for Orthopedic Implants Application. *Materials (Basel)* 14(19). doi: 10.3390/ma14195710.

Zhang, S., Cheng, X., Yao, Y., Wei, Y., Han, C., Shi, Y., et al. (2015). Porous niobium coatings fabricated with selective laser melting on titanium substrates: Preparation, characterization, and cell behavior. *Mater Sci Eng C Mater Biol Appl* 53**,** 50-59. doi: 10.1016/j.msec.2015.04.005.

Zhang, X., Li, J., Wang, X., Wang, Y., Hang, R., Huang, X., et al. (2018). Effects of copper nanoparticles in porous TiO2 coatings on bacterial resistance and cytocompatibility of osteoblasts and endothelial cells. *Mater Sci Eng C Mater Biol Appl* 82**,** 110-120. doi: 10.1016/j.msec.2017.08.061.

Zhang, X., Lv, Y., Fu, S., Wu, Y., Lu, X., Yang, L., et al. (2020b). Synthesis, microstructure, anti-corrosion property and biological performances of Mn-incorporated Ca-P/TiO(2) composite coating fabricated via micro-arc oxidation. *Mater Sci Eng C Mater Biol Appl* 117**,** 111321. doi: 10.1016/j.msec.2020.111321.

Zhang, Y.Y., Zhu, Y., Lu, D.Z., Dong, W., Bi, W.J., Feng, X.J., et al. (2021b). Evaluation of osteogenic and antibacterial properties of strontium/silver-containing porous TiO(2) coatings prepared by micro-arc oxidation. *J Biomed Mater Res B Appl Biomater* 109(4)**,** 505-516. doi: 10.1002/jbm.b.34719.

Zhao, J., Liu, Z., Ren, X., Wang, B., Cai, Y., Song, Q., et al. (2022). Coating-thickness-dependent physical properties and cutting temperature for cutting Inconel 718 with TiAlN coated tools. *J Adv Res* 38**,** 191-199. doi: 10.1016/j.jare.2021.07.009.

Zhao, Q., Yi, L., Hu, A., Jiang, L., Hong, L., and Dong, J. (2019). Antibacterial and osteogenic activity of a multifunctional microporous coating codoped with Mg, Cu and F on titanium. *J Mater Chem B* 7(14)**,** 2284-2299. doi: 10.1039/c8tb03377c.

Zhao, Q.M., Li, B., Yu, F.X., Li, Y.K., Wu, J.S., Peng, Z., et al. (2021). Cu-Co Co-Doped Microporous Coating on Titanium with Osteogenic and Antibacterial Properties. *J Biomed Nanotechnol* 17(7)**,** 1435-1447. doi: 10.1166/jbn.2021.3120.

Zhou, J., and Zhao, L. (2016a). Hypoxia-mimicking Co doped TiO2 microporous coating on titanium with enhanced angiogenic and osteogenic activities. *Acta Biomater* 43**,** 358-368. doi: 10.1016/j.actbio.2016.07.045.

Zhou, J., and Zhao, L. (2016b). Multifunction Sr, Co and F co-doped microporous coating on titanium of antibacterial, angiogenic and osteogenic activities. *Sci Rep* 6**,** 29069. doi: 10.1038/srep29069.

Zhou, W., Huang, O., Gan, Y., Li, Q., Zhou, T., and Xi, W. (2019). Effect of titanium implants with coatings of different pore sizes on adhesion and osteogenic differentiation of BMSCs. *Artif Cells Nanomed Biotechnol* 47(1)**,** 290-299. doi: 10.1080/21691401.2018.1553784.
